# Environmental Impacts and Life Cycle Assessment of Recycled Plastic Bricks

**DOI:** 10.1021/acsomega.6c04677

**Published:** 2026-09-08

**Authors:** Sirri Neba Nforsoh, Kayla Kurtz, Marlene Prien, Jitka Becanova, Simon Vojta, Rui Wang, Rakesh Paswan, Karla Pozo, Rainer Lohmann, Bradley Clarke, Sumanta Das, Vinka Oyanedel-Craver

**Affiliations:** † Department of Civil and Environmental Engineering, 4260 University of Rhode Island, 2 East Alumni Avenue, Kingston, Rhode Island 02881, United States; ‡ 54083 University of Rhode Island, Graduate School of Oceanography, 215 South Ferry Road, Narragansett, Rhode Island 02882, United States; § Australian Laboratory for Emerging Contaminants (ALEC), School of Chemistry, 200954 The University of Melbourne, Grattan Street, Melbourne, Victoria 3010, Australia; ∥ 28088 Universidad San Sebastián, Facultad de Ingeniería, Arquitectura y Diseño, Lientur 1457, Concepción, Bio-Bio 4080871, Chile; ⊥ Masaryk University, Faculty of Science, RECETOX, Kamenice 753/5, Brno 625 00, Czech Republic

## Abstract

This study evaluated the mechanical performance, contaminant release, and life-cycle impacts of recycled plastic blocks manufactured from beach-collected and facility-sourced plastic waste. Four formulations combining polyethylene terephthalate, high-density polyethylene, low-density polyethylene, sand, and glass were evaluated. Unweathered blocks had compressive strengths between 20.6–24.3 N/mm^2^, suitable for paving applications; however, higher plastic fractions led to lower strength and higher porosity. UV weathering reduced strength by 6–12% and increased metal leaching by up to ∼400%. Per- and polyfluorinated concentrations peaked at 13.8 ng/L; concentrations decreased slightly after weathering due to polymer photo-oxidation and embrittlement that enhanced sorption capacity and retention. Formulations with lower plastic content (∼33%) had higher strength and lower leaching. Life-cycle assessment identified extrusion and waste-plastic processing as dominant contributors to climate change (0.12 kg CO_2_-eq/kg block) and particulate matter (0.42 x 10^–3^ kg PM_10_-eq/kg block), driven by fossil-based energy use and process emissions. Blocks with higher plastic ratios exhibited greater environmental burdens, matching leaching trends. These findings revealed that incorporating waste plastics into construction materials can support waste management and infrastructure, but durability, contaminant release, and processing emissions are sensitive to the formulation. Optimizing plastic-to-aggregate ratios and improving processing efficiency can improve durability while reducing environmental and health risks.

## Graphical abstract

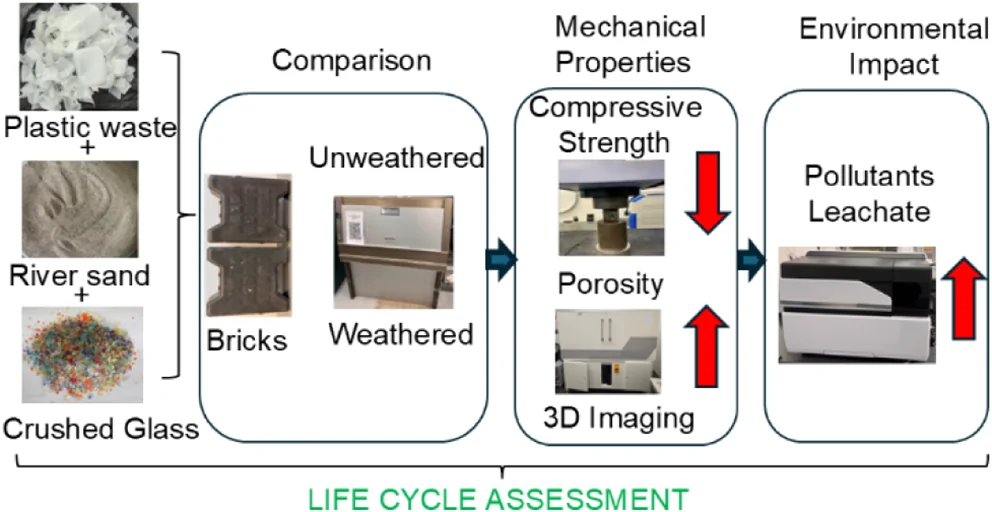

## 1 Introduction

Only about 9% of globally generated plastic waste is recycled, while 12% is incinerated, and 72% is sent to landfills or littered into the environment.^1^ Using recycled plastics in construction, such as bricks or pavers, has emerged as a waste-management strategy.^2^ Plastic bricks vary in strength depending on composition and fabrication method.^3,4^ Paving blocks are used for streets, sidewalks,^5^ or landscaping.^6^ Such initiatives are increasingly common globally.^7–9^

Recycling plastic waste into bricks involves several steps: collection of plastic waste, washing, sanitizing^10^ and drying.^2^ The dried plastic is shredded, melted, or extruded at temperatures between 105 °C and 260 °C.^6^ The blocks are cooled either in air at room temperature^11^ or in water.^3^ Studies reported that plastic bricks have compressive strengths, ranging from 4.95 to 61 MPa,^12,13^ with optimal strength at 30 to 40% plastic content.^14^

Plastics degrade in the environment through four primary mechanisms: photodegradation, thermooxidative degradation, hydrolytic degradation, and microbial biodegradation.^15^ This degradation reduces strength and produces brittleness, discoloration, and surface cracking.^16,17^ Additionally, they can leach chemicals, including manufacture additives, sorbed organic pollutants, and degradation products.^18^ While several studies have investigated the mechanical and physical properties of unweathered plastic bricks, limited research exists on the effects of weathering on the long-term performance of plastic bricks.^12,13^

The production and use of plastic bricks raise concerns about their potential impact on freshwater and marine ecosystems. Water usage during washing of waste plastics^19^ and cooling/curing stages^3^ may lead to the release of substances into water bodies. Elevated concentrations of polycyclic aromatic hydrocarbons (PAHs), polychlorinated biphenyls (PCBs), and polybrominated diphenyl ethers (PBDEs) have been detected in washing facilities of large-scale plastic recycling plants.^20^ Other studies have reported the presence of heavy metals in soil, sediment, and water samples near mechanical plastic waste recycling plants.^21^ Extrusion and shredding of plastic waste contribute to air pollution by emitting gases and particulate matter.^20,22^

Analyzing the environmental impacts of plastic block production through life cycle assessment (LCA) can be important to understanding the benefits and limitations. Previous studies have explored recycling, landfilling, and incineration of plastic waste, but most of them focus on recycling pathways rather than their use in construction applications. Amjad and Diaz-Elsayed (2024) investigated bricks made up of 100% waste plastic, with no aggregates added. The results showed considerable environmental benefits, including a 60% reduction in terrestrial ecotoxicity and a 28% reduction in water consumption.^23^

Previous studies have shown potential for utilizing plastic waste in brick manufacturing, mostly focusing on mechanical properties.^24^ However, less attention has been given to the effects of UV-induced degradation on the block performance and environmental impacts. This study addresses these gaps by evaluating plastic bricks manufactured from plastic waste in Timor-Leste, focusing on (1) the leaching potential of pollutants, (2) compressive strength, (3) the influence of UV weathering on leaching and strength, and (4) overall environmental performance through LCA.

## 2 Methods

This study was performed in three stages: (1) characterization: blocks from the manufacturer were prepared and analyzed for chemical composition; (2) performance testing: structural and environmental properties (compressive strength, porosity, leaching) were measured, with accelerated weathering used to simulate UV exposure; (3) LCA: environmental impacts were quantified following ISO 14040/44 across raw material extraction, processing, and end-of-life. This approach enabled direct comparison of formulations, integrating mechanical performance with environmental impacts. Figure 1 presents a visualization of the methods used.

**Figure 1:**
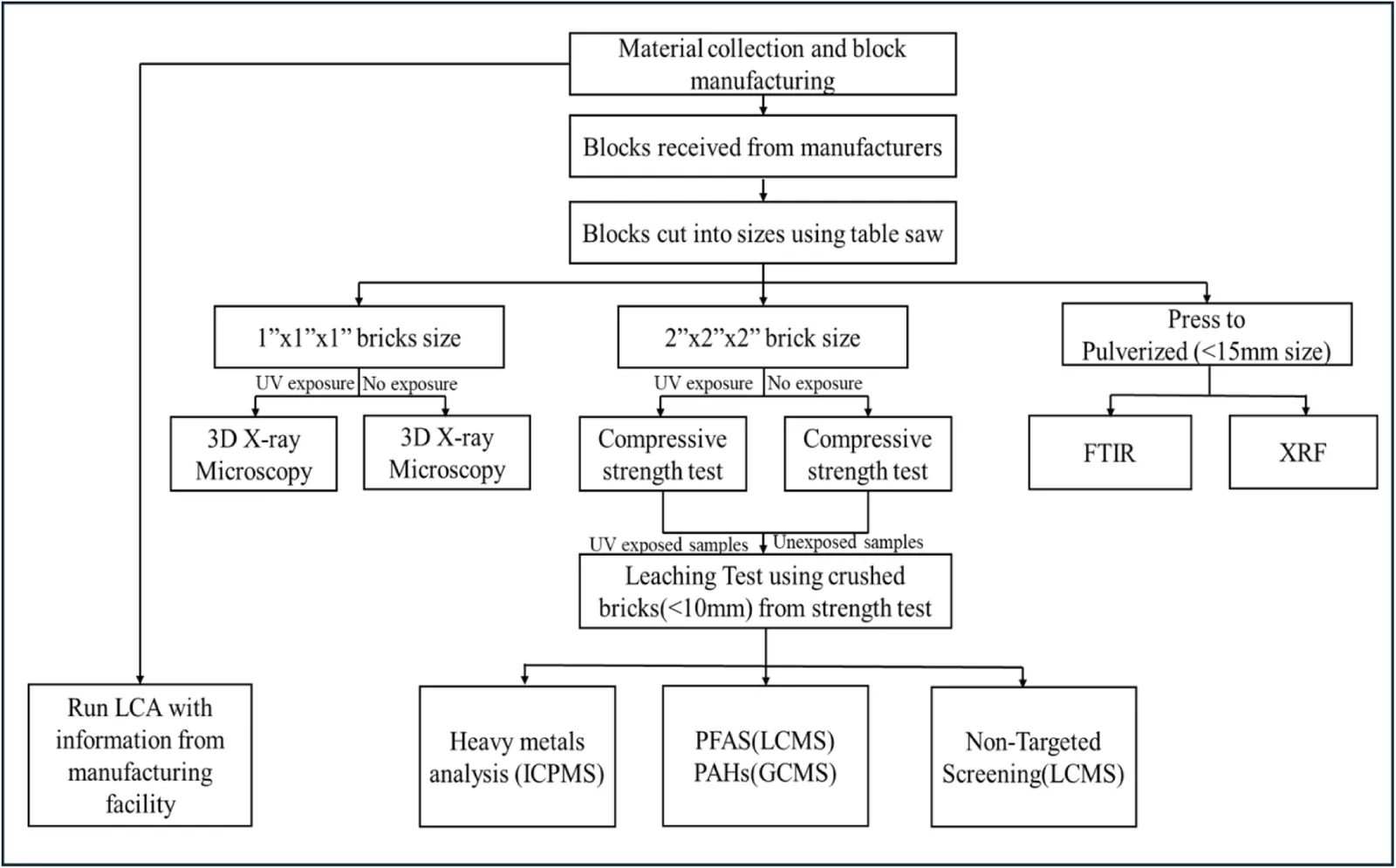
Schematic representation of the laboratory procedures and analytical methods used for the analysis of samples in this study.

### 2.1 Sample Preparation and Characterization

#### 2.1.1 Brick Manufacturing and Sample Preparation

Bricks were manufactured by a company in Timor-Leste. Plastic and glass waste were collected from beaches, community drop-off points, and partner organizations in Dili and transported to the company’s Material Recovery Center. Polyethylene terephthalate (PET), high-density polyethylene (HDPE), and low-density polyethylene (LDPE) were shredded into flakes; river sand was collected locally, dried, and sieved, while glass was pulverized into fine particles. Waste glass was chosen as a reinforcing material because of its availability as a waste material, its high silica content, chemical stability, stiffness, and low water absorption, which enhance the strength and durability of polymer composites while promoting the beneficial reuse of waste materials.^25,26^ Previous studies have reported that waste glass improves the mechanical performance and durability of construction materials while conserving natural resources and reducing environmental impacts.^25,27^

The prepared materials were mixed in specified proportions (Table 1) and extruded at 260–300 °C for ∼10 min. The resulting paste was molded into pavers using a hydraulic press, water-cooled, and stored. Some samples of the bricks are presented in Figure 2. An image of a brick (Figure S1) and more information on sample preparation is provided in Section S2 of the Supplementary information (SI).

**Table 1:** Compositions of Bricks Provided by the Company in Timor-Leste

	Compositions (% in weight)			
Material	1	2	3	4
Glass (Pulverized; 0-5 mm)	41.7	20.0	60.0	0.0
River sand/gravel (0-5 mm)	25.0	26.7	0.0	64.9
PET (Flakes; 5–7 x 5-7 mm)	8.3	13.3	13.3	8.1
HDPE (Flakes; 5–7 x 5-7 mm)	25.0	40.0	16.7	24.3
LDPE (Strips; 10 x 50 mm)	0.0	0.0	10.0	2.7
Total plastic percentage	33.3	53.3	40.0	35.1

**Figure 2:**
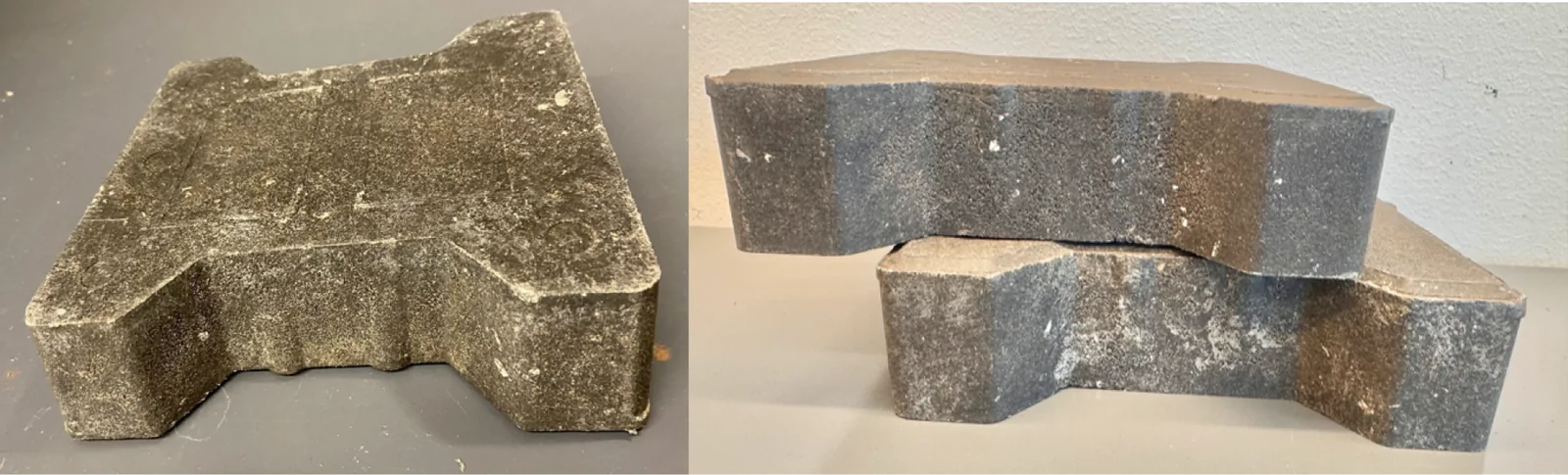
An actual image of the bricks.

#### 2.1.2 Accelerated Weathering

UV degradation testing was performed using a Q–U–V accelerated weathering tester (Q-Panel Company, Cleveland, OH, USA). The blocks were UV exposed for a duration of 500 h. Block samples of each composition (1 × 1 × 1 in. and 2 × 2 × 2 in. cubes) were placed in the weathering chamber under UVA-340 fluorescent lamps (Q-Lab, OH, USA) at 35–40 °C. The lamps provided an intensity of 0.7 W/m^2^/nm at 340 nm. An acceleration factor of ∼17 was considered to estimate the outdoor UV exposure time, and the Qingdao climate was used as stated by Lv et al. (2015), which is equivalent to ∼3–4 years of outdoor UV exposure.^28^ This represents ∼10% of the service life of paving blocks (40–50 years).^29^ After exposure, samples were stored at room temperature in the dark until analysis, same as the unexposed samples.

#### 2.1.3 Chemical Composition of Brick Samples

The elemental composition of the four brick formulations was analyzed using an energy-dispersive X-ray fluorescence spectrometer (EDXRF; Shimadzu EDX-8100, Shimadzu Corporation, Japan). The chemical composition was also determined using attenuated total reflectance Fourier-transform infrared spectroscopy (ATR-FTIR; Shimadzu IRTracer-100, Shimadzu Corporation, Japan) with LabSolutions IR software. More information on the elemental and chemical characterization is presented in S3 of the SI.

### 2.2 Performance of the Blocks

#### 2.2.1 Structural Performance Analysis

##### 2.2.1.1 Compressive trength Test

Unconfined compressive strength tests were performed on UV-exposed and unexposed 2 × 2 × 2 in. samples of all compositions following a standard method.^30^ Testing was conducted using a UH-600 kNX Universal Testing Compression Machine (Shimadzu Corporation, Japan) equipped with Shimadzu Trapezium software. A vertical load was applied at a constant rate of 1.4 N/s until specimen failure (Figure S2 of SI). All tests were conducted in triplicate for each composition.

##### 2.2.1.2 3D Imaging Analysis

Three-dimensional imaging of 1 × 1 × 1 in. UV-exposed and unexposed plastic brick samples were performed using a Zeiss Xradia 610 Versa X-ray Microscope (XRM) (Carl Zeiss X-ray Microscopy, Inc., Dublin, CA, USA). Duplicates of the same brick specimens were scanned before and after UV exposure. Data were acquired with Scout-and-Scan software at 29 μm voxel resolution, 160 kV operating voltage, 25 W power, 4× objective, 1 s exposure time, and no filter, under air atmosphere. Source-to-detector and source-to-sample distances were set to 182 mm and 133 mm, respectively. Images provide representative visualizations of the internal pore structure. Quantitative porosity measurements were obtained from the reconstructed XRM datasets using Dragonfly image analysis software through image segmentation and pore-volume analysis. The reported porosity values are based on these quantitative measurements, and porosity was then calculated as the ratio of the total segmented pore volume to the total analyzed specimen volume and reported as a percentage. To determine the damage and fracture mode of the block, the fracture surface of the specimen was imaged using a Zeiss Sigma VP Field-Emission Scanning Electron Microscope (FE-SEM, Cambridge, United Kingdom). The SEM operated at an accelerating voltage of 20.0 kV, and the fractured surfaces of the specimens were coated with a thin layer of gold prior to SEM analysis.

#### 2.2.2 Environmental Performance Analysis

Leachability of inorganic and organic contaminants from the plastic bricks was evaluated following the U.S. Environmental Protection Agency (EPA) Toxicity Characteristic Leaching Procedure (TCLP; Method 1311).^31^ The leaching solution was prepared using glacial acetic acid (≥99.7% w/w, Fisher Scientific, NJ, USA) and deionized water, adjusted to pH 2.88 ± 0.05. For each composition, 20 g of pulverized block material (<10 mm) was combined with 400 mL of leaching solution in an incubator shaker (Geno Technology Inc., St. Louis, MO, USA) and agitated at 150 rpm at 23 ± 2 °C for 18 ± 2 h.^32,33^ For inorganic analysis, leachates and controls (leaching solution without blocks) were collected in sterile 50 mL polypropylene tubes, acidified to pH < 2 with nitric acid, and stored at 4 °C before analysis. For organic analysis, leachates were collected in amber glass vials and stored at 4 °C in the dark for about 72 h prior to analysis.

Inorganic element concentrations in the leachates were measured by using inductively coupled plasma mass spectrometry (ICP-MS; Shimadzu ICPMS-2030, Shimadzu Corporation, Japan) following EPA Method 200.8. Operating conditions included a nebulizer gas flow rate of 0.70 L/min, an auxiliary gas flow rate of 1.10 L/min, a plasma gas flow rate of 10.0 L/min, a lens voltage of 7.25 V, and a radio-frequency power of 1200 W. All of the analyses were performed in triplicate. Additional details are provided in Section S5 of the SI.

PAHs in the leachates were analyzed using a gas chromatograph–mass spectrometer (GCMS-TQ NX Series; Shimadzu Corporation, Oregon, USA) operated with Shimadzu GCMS Real-Time Analysis software. Sixteen PAHs from the U.S. EPA priority pollutant list were targeted: acenaphthene, acenaphthylene, anthracene, fluoranthene, fluorene, naphthalene, phenanthrene, pyrene, benz­[a]­anthracene, benzo­[b]­fluoranthene, benzo­[k]­fluoranthene, benzo­[ghi]­perylene, benzo­[a]­pyrene, chrysene, dibenz­[a,h]­anthracene, and indeno­[1,2,3-cd]­pyrene.^34^ The analytical method was performed following the EPA method 8272 described by Cheng et al. (2013).^35^ All samples were analyzed in duplicate. Additional details on analytical conditions are provided in Section S6 of SI.

For PFAS, the leachate samples were extracted by solid-phase extraction (SPE). Duplicates of each composition were processed by SPE using a vacuum manifold (Sigma-Aldrich, MO, USA) with 250-mg hydrophilic-lipophilic balanced (HLB) cartridges (Waters Corporation, MA, USA). Cartridges were preconditioned with LC-MS-grade methanol and rinsed with LC-MS-grade water (Fisher Scientific, NJ, USA). Each sample (∼170 mL) was spiked with 4 ng PFAS surrogate standard, eluted with 4 mL of 1% ammonium hydroxide in methanol, and concentrated to 1 mL under nitrogen. The instrumental analysis of PFAS was performed using a SCIEX Exion UHPLC system coupled to a SCIEX X500R quadrupole time-of-flight tandem mass spectrometer (QTOF MSMS). The concentrated methanol tissue extract was reconstituted with 10 mM ammonium acetate in water to reach a final ratio of 40/60 (methanol/water). More instrumental analysis information is presented in Section S7 of SI. A total of 54 target PFAS were analyzed (full list of the compounds is presented in Table S8 of SI), but only those detected in at least one sample were reported. Reported concentrations were corrected using method detection limits determined from process blanks and expressed in ng/L, normalized to the analyzed volume.

For non-targeted organic analysis, leachates of aged and unaged compositions 3 and 4 were prepared following the method used by Peter et al. (2018).^36^ Pulverized block material (1000 mg, <10 mm) was mixed with 1 L of Milli-Q water and agitated for 48 h at 20 °C in an incubator shaker (Geno Technology Inc., St. Louis, MO, USA). Those two compositions were chosen based on their metal concentrations and compositions, which were very different. Leachate samples were stored at 4 °C for 2 days before extraction. Triplicates of each composition were processed by SPE. Final analyses were conducted at the Australian Laboratory for Emerging Contaminants (ALEC) on an Agilent 1260 Infinity HPLC coupled to an Agilent 6546 quadrupole time-of-flight high-resolution mass spectrometer (QTOF-HRMS). More information on SPE extraction and analytical parameters is presented in Section S9 of SI.

### 2.3 LCA Methodology

LCA was conducted in accordance with ISO 14040 and 14044 guidelines. The goal and scope phases defined the study purpose, life cycle stages, and system boundaries. The life cycle inventory (LCI) quantified material and energy inputs as well as emissions, while the life cycle impact assessment (LCIA) evaluated potential impacts using the ReCiPe midpoint (H) v1.13 method with a 100-year time horizon. Eighteen midpoint impact categories were considered (Table S2 of the SI), including climate change potential (GWP100), human toxicity potential (HTPinf), marine eutrophication potential (METPinf), and particulate matter formation potential (PMFP), which were selected for detailed analysis because they were the four most relevant impact categories for the study area since it is a coastal area. Finally, in the interpretation phase, the LCI and LCIA results were synthesized to identify key inputs, outputs, and potential environmental impacts of the product or service.^37^

The analysis was conducted in two parts: (1) ranking the four block formulations across all 18 impact categories, and (2) identifying the top three contributing processes to GWP100, HTPinf, METPinf, and PMFP using contribution analysis. These categories were prioritized given the regional vulnerability of Timor-Leste to climate change,^38^ the potential for weathered plastics to generate microplastics harmful to the marine system,^39^ and the release of volatile substances during plastic extrusion, posing health and air quality risks.^40^

The functional unit (FU) was defined as the production of 1,000 pavers (7.87 × 6.69 × 1.97 in.; 2.1 kg each). Impacts from raw material supply and manufacturing were assessed. Due to limited site-specific data, vegetable oil was modeled as the mold release agent, and hydraulic press energy demand was estimated from fired-clay brick production. The Ecoinvent 3.8 database was used, and calculations were performed in Brightway 2.4.1. Additional inventory details are provided in Table S3 of SI.

## 3 Results and Discussion

### 3.1 Chemical Composition of Brick Samples

Elemental composition results for all four formulations are summarized in Table S4 (SI). Si, K, Ca, Al, Ti, Cr, Mn, Fe, Cu, and Zn were detected in all blocks, primarily reflecting contributions from sand and glass.^41^ Pb and Sr appeared in 3 formulations, while Br, Ba, and V were found in two. Br likely originates from brominated flame retardants,^42^ whereas Cr, Pb, Cu, Ni, and related metals are associated with manufacturing additives commonly found in recycled plastics, such as inorganic pigments, UV and heat stabilizers, and antioxidants.^42,43^

FTIR spectra for all compositions (Figure S3, SI) confirmed the presence of PET, HDPE, LDPE, sand, and glass. Peak assignments matched known reference spectra from previously reported peak data in the literature, validating the manufacturer-reported material composition (Table S5, SI).

### 3.2 Performance Analysis

#### 3.2.1 Structural Performance Analysis

The compressive strength test results for bricks before and after UV exposure are presented in Figure 3.

**Figure 3:**
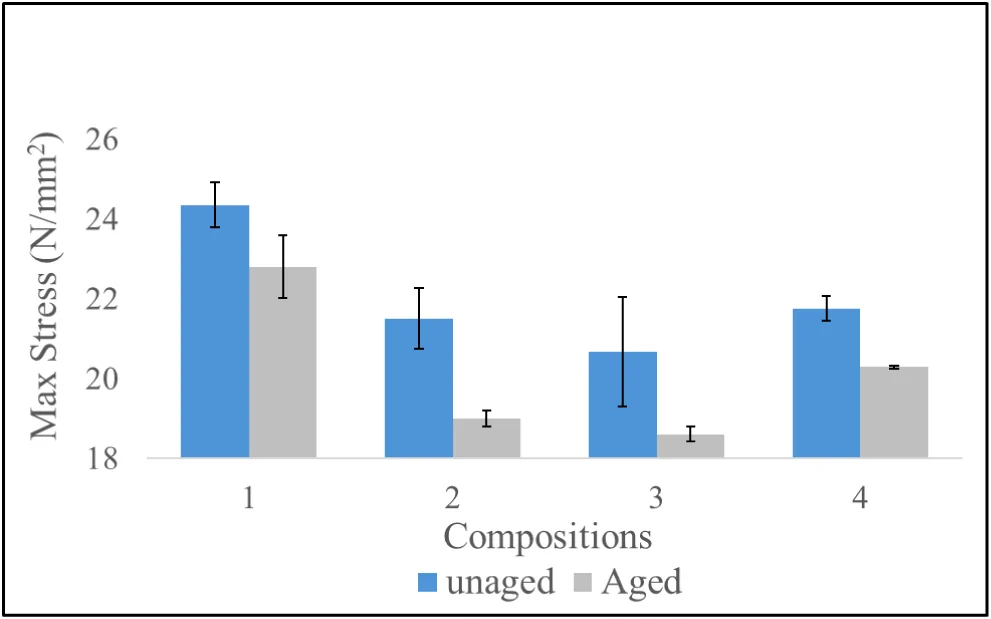
Compressive strength for unaged and aged plastic blocks (Compositions 1–4). Bars represent mean compressive strength with standard deviations.

Compressive strength results showed significant variation (p = 0.003) among the four formulations, with unaged blocks ranging from 20.6 ± 1.37 to 24.3 ± 0.57 N/mm^2^. The high strength of Composition 1 can be attributed to the viscoelastic behavior of PET at high temperatures and the reinforcing effect of crushed glass, which together reduced porosity (porosity results are described below) and increased density (Figure S7 of SI).^10^ Composition 1, with ∼33% plastic content, also showed the lowest porosity, consistent with prior studies indicating that ∼30% plastic to 70% aggregate yields optimal strength, whereas higher plastic fractions reduce performance.^44,45^ UV exposure reduced strength by 6–12% for all formulation, with statistically significant differences among the samples (p < 0.001), and between the aged and unaged sets (p = 0.02). Greater strength losses in higher-plastic-content blocks (Compositions 2 and 3) reflect the response of polymers to UV-induced degradation.^46^ In contrast, formulations with higher proportions of aggregates (sand and glass) retained strength more effectively (Compositions 1 and 4).

3D imaging showed pores of varying sizes in all formulations. Figures S4–S5, SI present representative XRM images for both the unexposed and UV-exposed block samples, with cracking most apparent in the high-plastic Composition 2. Although pores are visible in both specimens, the increase in total porosity by 6–10% across compositions following UV exposure was determined from quantitative image segmentation and pore volume analysis performed using Dragonfly software, rather than from visual inspection of the XRM images alone. The representative images are provided to illustrate the internal pore morphology, while the reported porosities for each composition presented in Figure 4 are based on quantitative analysis of the reconstructed datasets. An example of the 2D and 3D views of the segmented pores is presented in Figure S6, SI.

**Figure 4:**
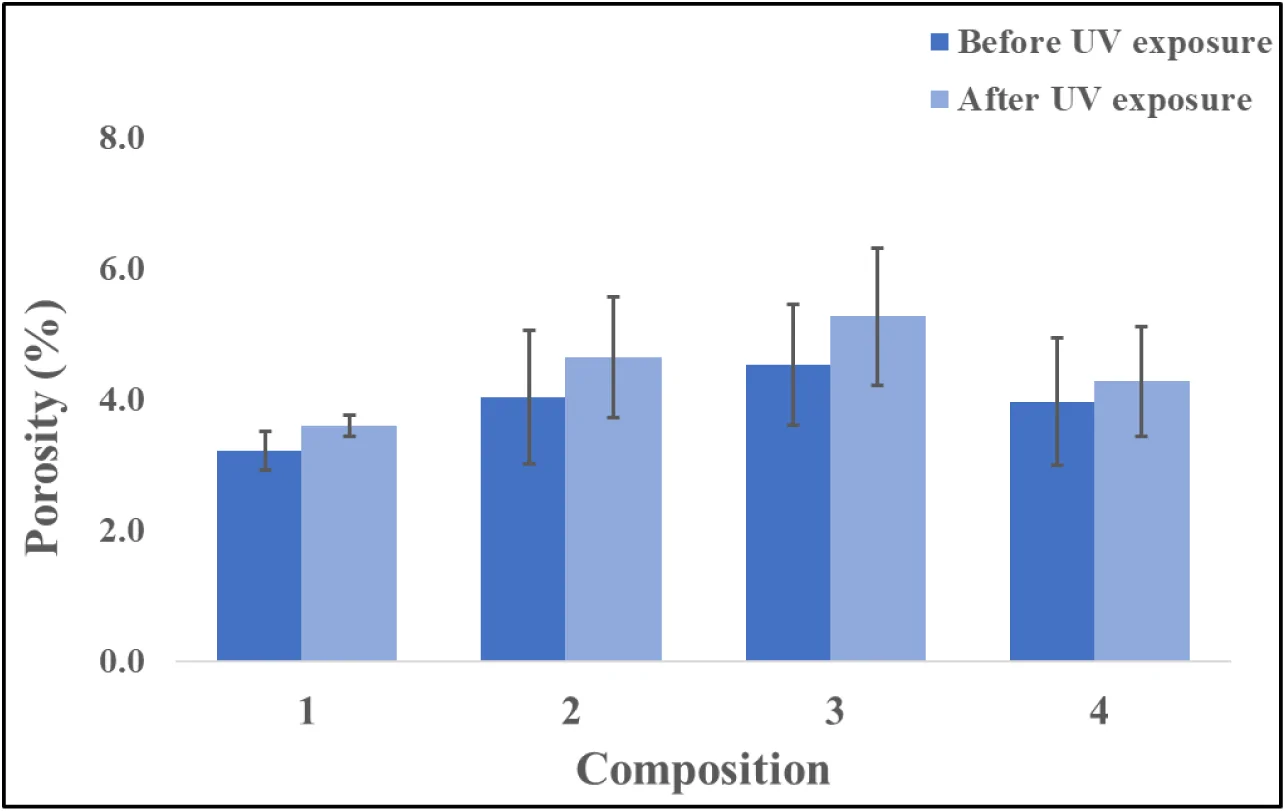
Porosity of the different block compositions for the unexposed and UV-exposed block samples was calculated from the quantification results as the percentage of pores in each sample relative to the total volume of the 1-inch blocks.

Total porosity in unexposed samples ranged from 3.2–4.5%, with composition 3 having the most pore volume. The pores observed in the bricks can be likely attributed to a combination of manufacturing-related mechanisms. During melting and mixing, air can become entrapped within the highly viscous polymer melt and remain trapped during solidification, particularly when there is insufficient time or pressure during molding for complete air evacuation.^47,48^ In addition, volumetric shrinkage of the polymer during cooling may generate internal shrinkage cavities, further contributing to porosity.^47^ These manufacturing-induced pores act as stress concentration sites that facilitate crack initiation and reduce the mechanical performance of polymer composites.^49^ From the quantitative analysis, total porosity increases in UV-exposed samples. This increase can be attributed to UV-induced degradation of the polymer matrix, leading to structural breakdown of the polymer and pore formation.^50^ Pore-volume distributions (Figure S7, SI) were dominated by pores of <0.5 mm^3^. Overall, porosity results align with the observed UV-related decreases in compressive strength.

The relationship between compressive strength and the porosity of the blocks for compositions 1–4 is presented in Figure 5.

**Figure 5:**
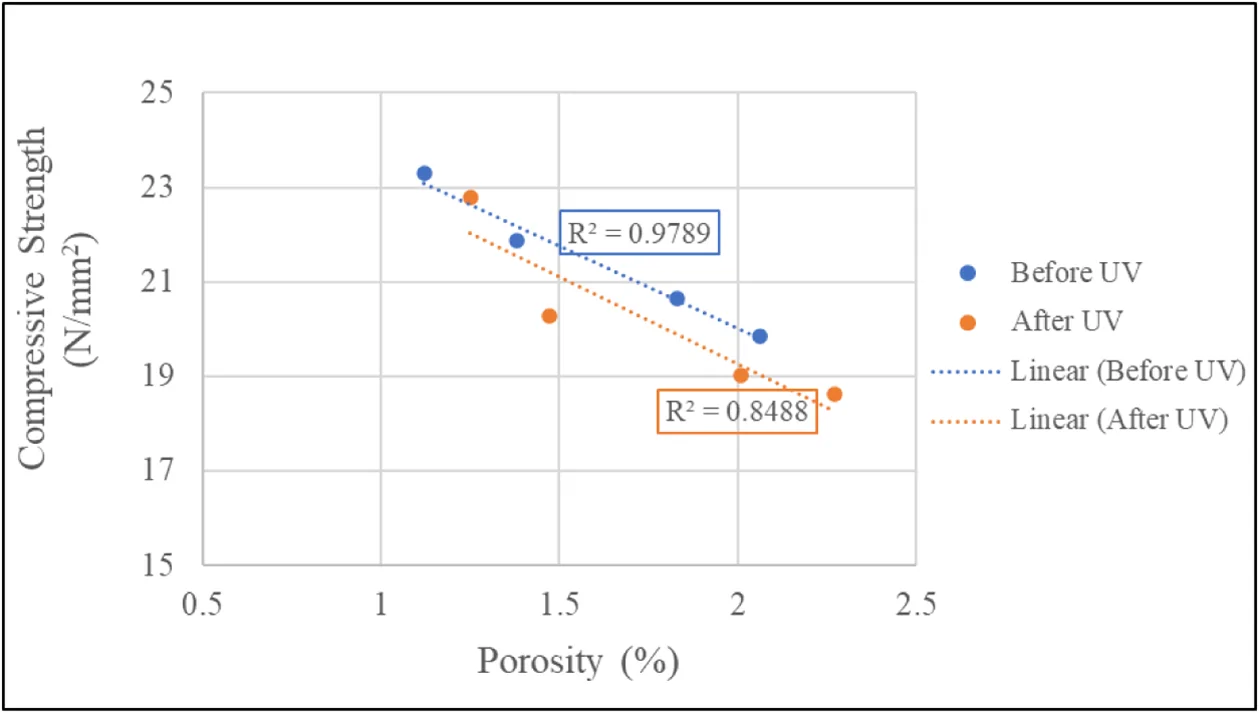
Compressive strength vs porosity for all compositions before and after UV exposure. Each point represents one composition (n = 4 per condition). Solid lines are linear regression fits.

An inverse relationship was observed between porosity and compressive strength before and after UV exposure, as higher pore content reduced the load-bearing area and introduced stress concentrators, leading to strength loss.^51^ After UV aging, a lower R^2^ indicated added damage from polymer embrittlement and interfacial degradation.^52,53^ Composition 1 had the lowest porosity and highest strength, likely due to well-graded aggregates and moderate plastic content. Composition 2 showed higher porosity and lower strength, consistent with reduced mineral bonding at higher plastic fractions.^12,54^ Composition 3 had the highest porosity and lowest strength, likely from poor packing and voids around LDPE fragments.^55^ Composition 4 showed low porosity and intermediate strength, reflecting efficient, sand-dominated compaction with moderate plastic content.^56^

The fracture mechanism of the plastic-sand-glass composite was governed by progressive damage involving polymer deformation, interfacial debonding, particle pull-out, and crack coalescence, as shown in Figure 6.

**Figure 6:**
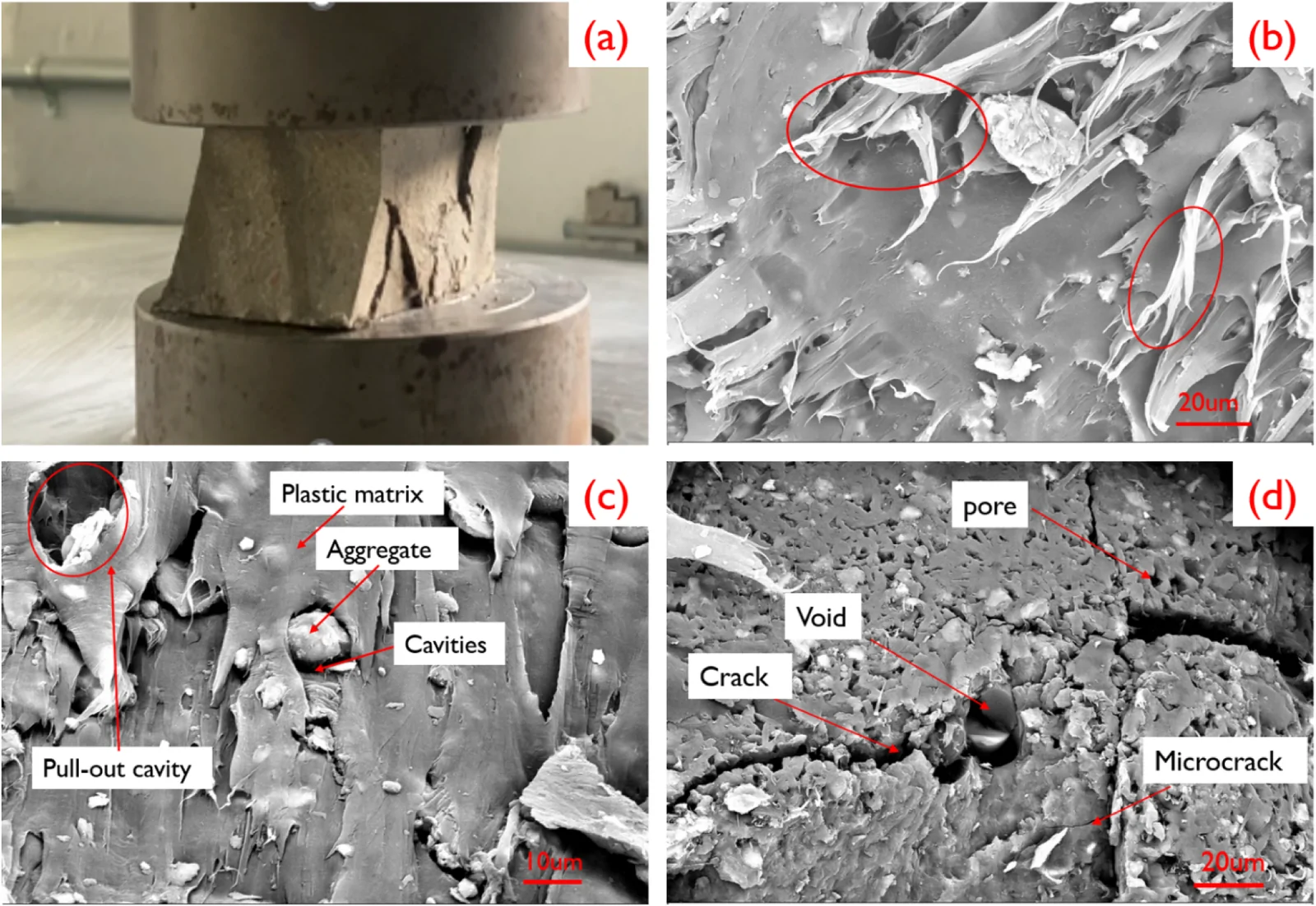
Fracture characteristics of the block after compressive loading. (a) Macroscopic specimen image showing the inclined shear fracture plane formed during compressive failure. (b) SEM image showing polymer fibrils, torn ligaments, and ductile deformation of the thermoplastic binder. (c) SEM image showing particle pull-out, interfacial separation, and voids surrounding sand and glass particles or aggregates. (d) SEM image showing microcracks, pores, crack coalescence, and the final fracture morphology.

The macroscopic image Figure 6a shows a distinct inclined diagonal fracture plane extending across the specimen, indicating that failure occurred predominantly by shear under compressive loading. The SEM images provide direct evidence of the associated micromechanical damage. Figure 6b shows elongated polymer fibrils and torn ligaments spanning the fracture surface, indicating that the thermoplastic binder underwent substantial plastic deformation and ductile tearing before rupture. Such fibrillar fracture features are characteristic of energy dissipation through plastic deformation during the fracture of thermoplastic polymers.^57^ Figure 6c shows cavities surrounding sand and waste glass particles, suggesting particle–matrix interfacial debonding associated with localized stress concentrations arising from the stiffness mismatch between the ductile polymer binder and the rigid mineral aggregates. Particle pullout was also observed, indicating localized separation of the polymer binder from the aggregates. Such interfacial defects reduce the efficiency of stress transfer between the binder and the aggregates and act as preferential sites for crack initiation and propagation, consistent with established damage mechanisms in particle-reinforced polymer composites.^58,59^ Figure 6d shows nterconnected microcracks and pores, indicating that cracks initiated at interfacial defects and existing voids before progressively coalescing into the macroscopic fracture plane. Similar fracture mechanisms have been reported for plastic-bonded sand composites, where inadequate interfacial bonding and porosity promote crack initiation and reduce compressive strength.^60^ Overall, the fracture surfaces indicate that failure occurred through a mixed fracture mechanism, involving ductile deformation of the thermoplastic binder together with interfacial damage, particle pullout, and progressive microcrack coalescence, leading to compressive failure.

#### 3.2.2 Environmental Performance Analysis

From all compositions analyzed (1, 2, 3, and 4), metals including Al, Cr, Cu, Fe, Mn, Pb, Ti, and Zn were detected in leachates from all block formulations, as seen in Figure 7, consistent with XRF analysis results.

**Figure 7:**
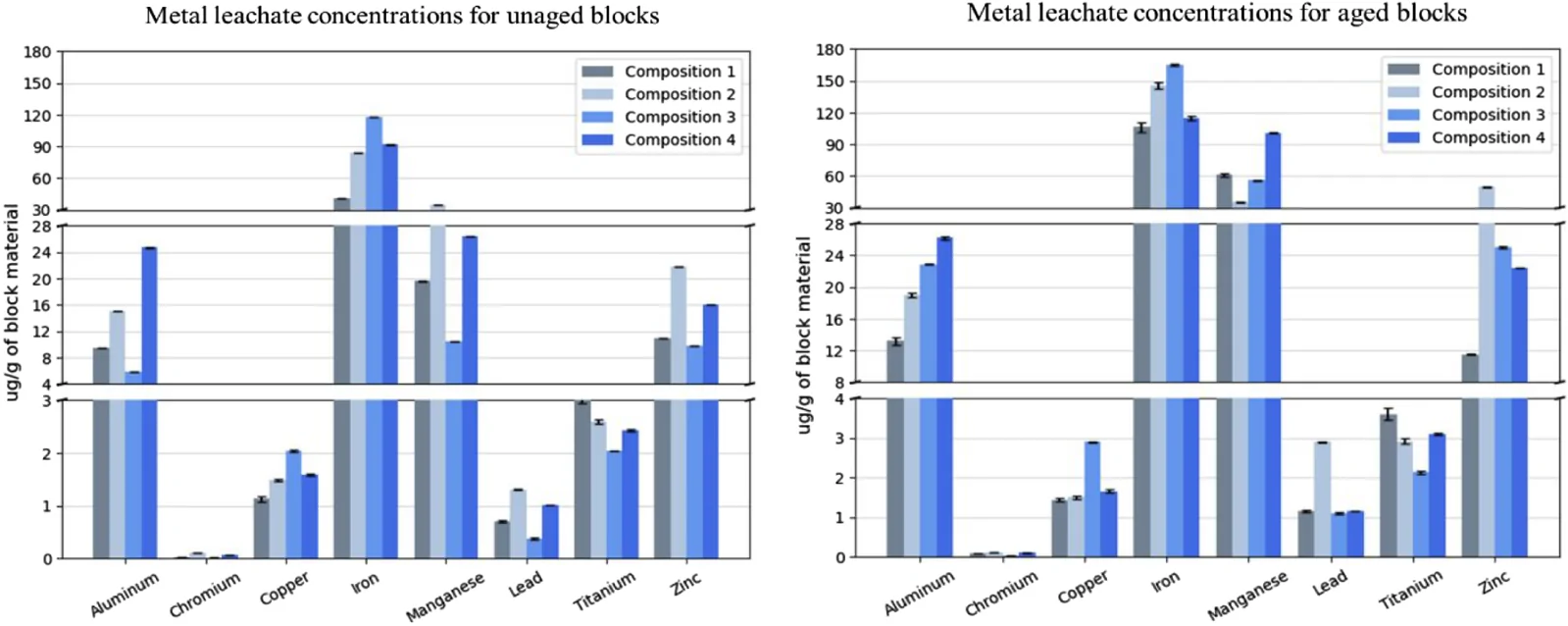
Leachate metal concentrations for unaged and aged plastic blocks (Compositions 1–4). Stacked bars represent mean metal concentrations. Relative to pre-UV, the post-UV distribution shifts toward higher metal concentrations.

The concentrations of all metals varied significantly across compositions (p < 0.001). Compositions 2 and 4 showed higher levels for most metals, likely due to metal-containing plastic additives.^42^ Composition 3 (60% glass) generally released lower metal levels, except for Fe, consistent with glass acting as a partial diffusion barrier.^61^ Higher Fe and Al in Composition 4 align with its high sand content.^62^ Variations in material ratios prevented a single leaching pattern. After UV exposure, metal concentrations increased significantly (p < 0.0097), in some cases up to ∼400% compared to unexposed samples. UV exposure increased metal release due to polymer oxidation, embrittlement, and greater exposed surface area.^63^ Higher porosity after weathering may also contribute, though this cannot be confirmed without additional analysis.

Average concentrations of PAHs in block leachates, before and after UV exposure, are presented in Table S6 of SI along with detection and quantification limits. All 16 priority PAHs were below detection or quantification limits in both unexposed and UV-exposed samples, indicating negligible leaching. PAHs are not expected in these formulations because they are not intentionally added to plastic formulations during manufacturing, but are formed from combustion sources.^64^ Low levels likely reflect pre-cleaned feedstock that removed surface-bound PAHs acquired from environmental exposure, the absence of PAH-bearing additives (e.g., carbon black, extender oils), and extrusion temperatures too low for PAH formation.^65,66^

PFASs were detected in all samples. Of the 54 compounds screened, 14 were identified in at least one composition, as presented in Figure 8.

**Figure 8:**
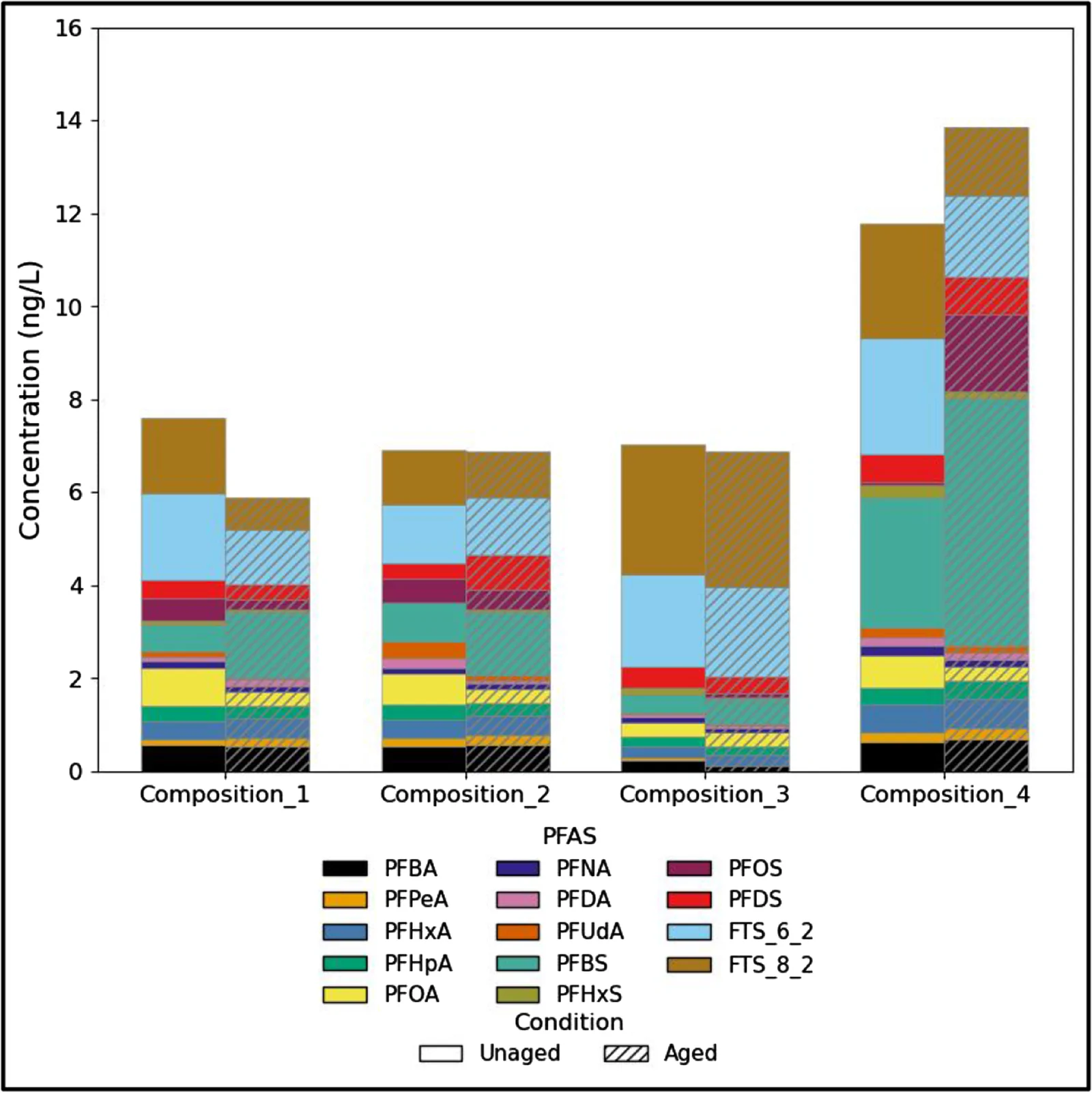
Leachate concentrations for PFAS compounds of unaged and aged plastic blocks (Compositions 1–4). Stacked bars represent mean PFAS concentrations.

For unaged samples, Composition 4 showed higher concentrations of most of the PFAS compounds. Sum of PFAS concentrations (ΣPFAS) ranged between 6.9 ng/L and 11.8 ng/L in the unexposed samples. Significant differences were observed among the four compositions (p<0.05) for all PFAS compounds except PFNA, PFDA, and PFUdA. The presence of PFAS in the samples is most likely due to environmental sorption of PFAS to materials prior to collection,^67^ especially from beaches, where hydrophilic and hydrophobic interactions facilitate PFAS binding to polymer surfaces,^68,69^ and contamination during the manufacturing process of the blocks. Concentrations were lower than those previously reported for beached pellets.^68^ For UV-aged samples, Composition 4 showed the highest leachate concentrations for most of the PFAS compounds. The concentrations of total PFAS slightly decreased in the UV-exposed samples by percentages ranging from 1.4% to 23.7%, except for Composition 4 which increased by 17.7%. The change in concentrations between aged and unaged samples was significant for selected PFAS, mainly for PFOA, PFBS, PFHxS, PFOS, and FTS species (p < 0.05), suggesting that aging processes could influence their release. The observed decrease in PFAS concentrations in the leachates after UV aging may be attributed to polymer oxidation and structural changes, which increase surface area and roughness, improving sorption capacity, and retention of PFAS,^70^ hence limiting their release into the leaching solution. PFAS such as PFNA, PFDA, PFUdA, and PFDS were less affected by aging, implying stronger sorption or lower vulnerability to aging-related changes. Table S7 provides full PFAS profiles.

Principal component analysis (PCA) was used to examine the correlations among material composition variables (sand, glass, and plastic), PFAS, and metal concentrations in the four compositions of unaged samples. Only the compounds and metals that were significantly different across compositions were included in the PCA. The first two principal components (PC1 and PC2) explained 82.5% of the overall variation, as presented in Figure S8 of SI. PFAS species clustered along PC2, showing strong correlations with Composition 4, which revealed higher PFAS concentrations. PFASs were correlated with higher sand content and lower plastic/glass content.^71,72^ In contrast, metals aligned with high plastic content, are consistent with metal-containing additives in plastics.

The concentration differences between aged and unaged (Δ = aged – unaged) samples were also used on another PCA, and only compounds that showed significant differences after aging were considered, which is presented in Figure S9 of SI. Compositions rich in sand (1 and 4)­were associated with increased PFAS (PFBS, PFOS) and metals (Cr, Ti, Mn) leaching, while plastic and glass-rich composites (Compositions 2 and 3) showed reduced changes after aging. Sand-rich matrices showed a greater increase, likely due to lower sorption capacity and UV-induced microcracking.^71^ Aged polymers develop oxidized functional groups that promote PFAS retention through adsorption and diffusion entrapment.^73^ Overall, aging increased composition-dependent contaminant behavior.

The non-targeted screening conducted on the plastic leachates for aged and unaged samples of compositions 3 and 4 demonstrated the presence of organic additives frequently used in plastics to enhance their properties, probably because many plastic additives are not covalently bound and can migrate into contacting media.^74^ The additive groups identified are mostly plasticizers, stabilizers, flame retardants, and antioxidants, as presented in Table 2.

**Table 2:** Identified Plastic Additives and Compounds by Non-Targeted Screening in Leachate Samples

Name	Formula	Composition	Compound Group	References
Niacinamide	C6H6N2O1	Composition 4	Cosmetics	^75^
Laurylethanolamide	C14H29N1O2	UV Composition 4	Cosmetics	^76^
Benzaldehyde	C7H6O1	Composition 3	Plasticizer	^77^
Galaxolidone	C18H24O2	Composition 4	Personal care products	^78^
Tris(2-butoxyethyl) phosphate	C18H39O7P1	UV Composition 4	Plasticizer, flame retardant	^77,79^
Tranexamic acid	C8H15N1O2	UV Composition 3	pharmaceuticals	^80^
6-Phenyl-1,3,5-triazine-2,4-diamine	C9H9N5	Composition 4	Antioxidant	^77^
Scopoletin	C10H8O4	UV composition 4	pharmaceuticals	^81^
Triethyl citrate	C12H20O7	UV composition 4	Plasticizer	^82,83^
DEET	C12H17N1O1	Composition 4	Insecticides	^84^
5-Hydroxyculmorin	C15H26O3	UV Composition 4	NA	
Triphenylphosphine oxide	C18H15O1P1	UV composition 4	Flame retardant	^85^
Benzaldehyde, 3-hydroxy-	C7H6O2	Composition 4	NA	
Monoethyl phthalate	C10H10O4	UV Composition 4	Plasticizer	^79^
Diethyl phthalate	C12H14O4	UC Composition 3	Plasticizer	^42,77^
Propylparaben	C10H12O3	UV Composition 4	Preservative	^86^

These detections corroborate that additives are mobilized under the applied leaching conditions, in line with recent evidence that plastic products leach diverse chemicals^74^ that can potentially affect the environment. Other organic compounds were also detected, which are not principal plastic additives, and their presence may be attributed to contamination arising from inadequate cleaning of the plastic containers before use. For example, residues from previously stored materials, such as edible oils, detergents, cleaning agents, or cosmetic products, can remain adsorbed on the container surface, even after rinsing, and may be detected during chemical analysis. More information about the compounds is presented in Table S8 of the SI.

### 3.3 LCA Results

The results from the LCA, ranking the impacts of the different compositions with respect to impact categories, are presented in Table 3.

**Table 3:** Ranking of the Impact of the Four Compositions from Low =1 to High = 4, for Each Impact Indicator^a^

Impact categories	Composition			
1 = lowest environmental impact (harm/benefit)	C1	C2	C3	C4
4 = highest environmental impact (harm/benefit)				
ALOP^b^	1	4	2	3
FDP, FEP, FETPinf, GWP100, HTPinf, IRP_HE, MDP, MEP, METPinf, ODPinf, PMFP, POFP, TAP100, ULOP, WDP^c^	1	4	3	2
NLTP^b^	1	4	3	2
TETPinf^b^	2	4	3	1

a The numbers were assigned based on the values gotten for different processes for each indicator, and four iterations were performed for each and ranked.

b The ranking of the impact of the different compositions varies for ALOP, NLTP, and TETPinf.

c For most impact categories, the compositions have the same ranking in terms of their impact.

For 16 out of 18 impact categories, composition 1 resulted in the lowest environmental harm and the highest benefit for natural land transformation potential (NLTP). Its environmental impact for urban land occupation potential (ULOP) was also low. In contrast, Composition 2 showed the highest environmental harm across most categories. Compositions 3 (60% glass) and 4 (no recycled glass) produced moderate impacts, suggesting that glass is not a significant driver of environmental impact, indicating limited influence of glass on overall impacts. Thus, it appears that higher plastic content leads to both increased environmental harm and benefits, since the majority of the impact categories were ranked 4. A higher plastic content consistently increased environmental burdens.

To identify areas with activities and processes that offer the greatest potential for improvement, a contribution analysis was conducted. Focusing on the context of Timor-Leste and the examined product, the impact categories selected for this analysis were GWP100, HTPinf, METPinf, and PMFP, as they appear to be the most important impact categories for the specific region. Figure 9 shows the most contributing activities for these four impact indicators.

**Figure 9:**
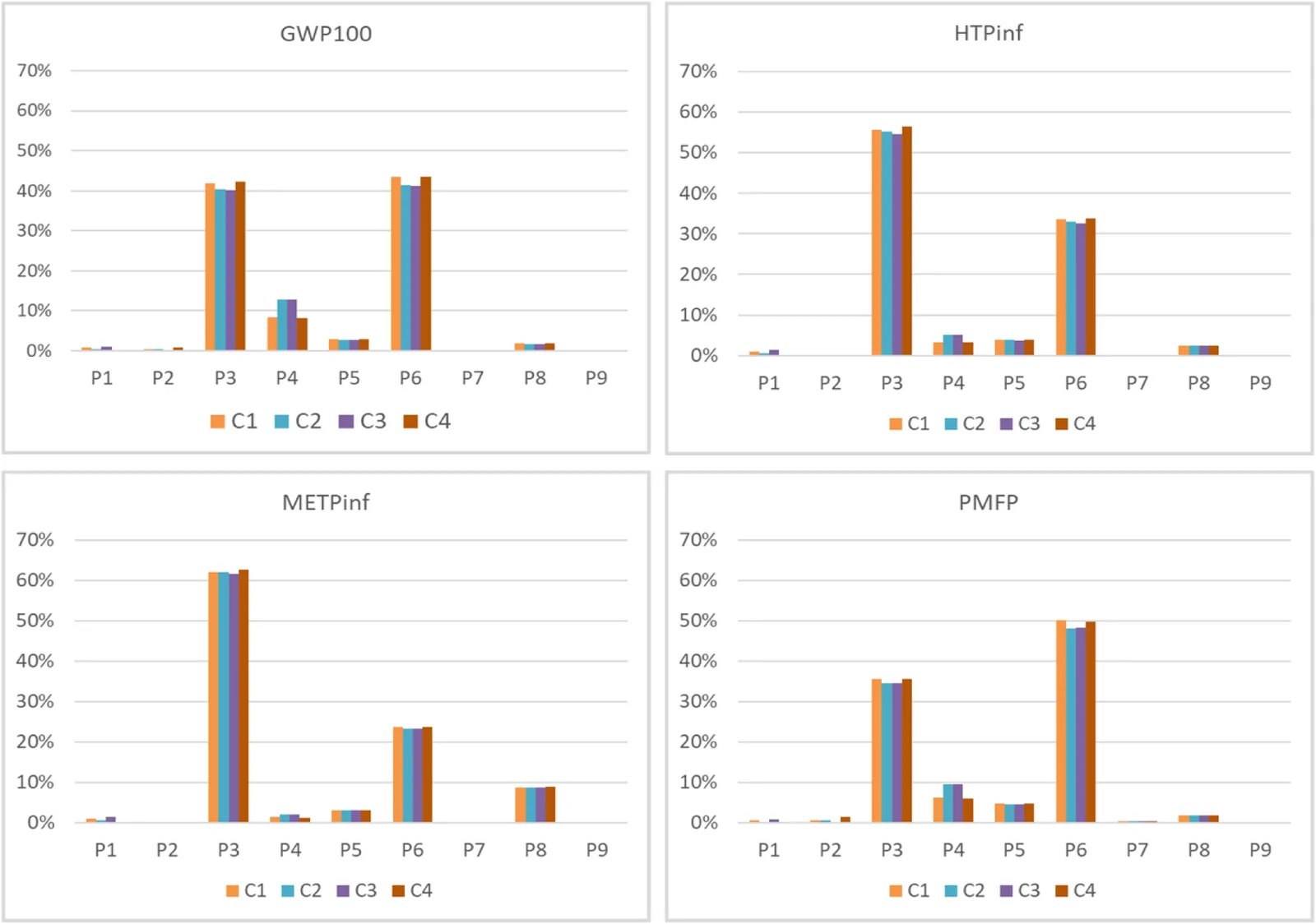
Breakdown of the environmental impact contributions by different activities P1–P9 to Climate Change (GWP100), Human Toxicity (HTPinf), Marine Ecotoxicity (METPinf), and Particulate Matter Formation (PMFP). The raw material supply and manufacturing of the blocks include the following processes: P1: Processing of waste glass; P2: Sand extraction; P3: Processing of waste plastic; P4: Drying of recycled PET; P5: Mixing raw materials; P6: Extrusion; P7: Electricity for the hydraulic press; P8: Mold release; P9: Water for cool-down.

Across all compositions, extrusion and waste plastic processing dominated impacts across the four categories, with a third major contributor varying by category (PET drying, raw material mixing, or vegetable oil production). Details of the three top contributing processes for each impact category are provided in Tables S9–S13 of SI. Although waste plastic processing contributed most to HTPinf, the primary process within this activity contributed slightly less than extrusion, suggesting that multiple subprocesses in waste plastic processing collectively drive its high toxicity impact. The total impacts of the categories for each composition are also presented in Table 4.

**Table 4:** Total Impacts and Most Contributing Activities to GWP 100, HTPinf, METPinf, and PMFP for Each Composition

Impact Category	Compositions	Total impact	Main contributors
GWP 100 (kg CO_2_-eq)	C1	586.73	Plastic processing (41.95%) and extrusion (43.57%)
	C2	610.40	Plastic processing (40.32%) and extrusion (41.41%)
	C3	612.29	Plastic processing (40.18%) and extrusion (41.27%)
	C4	581.91	Plastic processing (42.30%) and extrusion (43.62%)
HTPinf (kg 1.4-DCB-eq)	C1	98.17	Plastic processing (55.70%) and extrusion (33.62%)
	C2	99.11	Plastic processing (55.17%) and extrusion (32.92%)
	C3	100.02	Plastic processing (54.67%) and extrusion (32.63%)
	C4	96.85	Plastic processing (54.67%) and extrusion (32.63%)
METPinf (kg 1.4-DCB-eq)	C1	1.10	Plastic processing (62.06%) and extrusion (23.65%)
	C2	1.10	Plastic processing (62.10%) and extrusion (23.39%)
	C3	1.11	Plastic processing (61.58%) and extrusion (23,20%)
	C4	1.09	Plastic processing (62.73%) and extrusion (23.73%)
PMFP (kg PM10 -eq)	C1	1.79	Plastic processing (35.50%) and extrusion (50.10%)
	C2	1.84	Plastic processing (34.55%) and extrusion (48.20%)
	C3	1.84	Plastic processing (34.57%) and extrusion (48.23%)
	C4	1.79	Plastic processing (35.60%) and extrusion (49.88%)

The LCA results show only minor differences in the total impacts among the four compositions. GWP100 ranged from 581.91 to 612.29 kg CO_2_-eq, HTPinf from 96.85 to 100.02 kg 1,4-DCB-eq, METPinf from 1.09 to 1.11 kg 1,4-DCB-eq, and PMFP from 1.79 to 1.84 kg PM_10_-eq. Across all impact categories, plastic processing and extrusion were the dominant contributors, accounting for more than 80% of GWP, ∼88% of HTPinf, ∼85% of METPinf, and over 83% of PMFP. The GWP 100 results are higher than the ones reported by Goyal et al. (2023), which is 9.86 kg CO_2_-eq m^–2^ for plastic-binder pavers.^87^ Also, the environmental impact trends observed in the present study are consistent with those reported by Budihardjo et al. (2026) for recycled PET-sand paving blocks.^88^ Only slight differences were found in environmental impacts between the 29:71 and 27:73 PET:sand mixtures, with the 27:73 ratio exhibiting lower impacts across all categories. For example, marine aquatic toxicity decreased from 89.26 to 78.00 kg 1,4-DCB-eq, indicating that reducing the PET content improved environmental performance.^88^ Similarly, the present study showed only minor variations among the four composite formulations, with METPinf from 1.09 to 1.11 kg 1,4-DCB-eq; however, the present study had lower impact values. In all the studies, the environmental burdens were dominated by the energy-intensive thermal processing stage, demonstrating that manufacturing energy consumption has a greater influence on life cycle impacts than relatively small changes in material composition^87,88^ and can be identified as one major lever to reduce the environmental burdens.

Processing of waste glass, sand extraction, electricity for the hydraulic press, and water for cooling were the least contributors to each impact category, contributing less than 3% of the impacts for all compositions. The low contribution of these processes to all impact categories can be attributed to their relatively low energy and material requirements compared with the energy-intensive plastic processing and extrusion stages. Recycled glass requires limited reprocessing, while sand extraction generally has a lower environmental burden than that of thermally intensive manufacturing processes. Likewise, hydraulic pressing and cooling consume substantially less electricity than extrusion, which involves heating and melting of the plastic. Similar findings have been reported in previous LCA studies, where energy-intensive manufacturing processes such as extrusion were identified as the dominant environmental hot spots,^87,88^ whereas recycled aggregate preparation and auxiliary operations contributed only marginally to the overall impacts. Likewise, Tushar et al. (2023) demonstrated that recycled glass processing through simple crushing has substantially lower environmental impacts than those of more energy-intensive manufacturing operations.^89^ Although direct numerical comparisons should be interpreted cautiously because of differences in functional units and system boundaries, these studies collectively support the present findings that the environmental performance of recycled plastic paving blocks is governed primarily by energy-intensive manufacturing processes rather than by modest variations in material composition.

Contribution analysis also revealed that thermal and mechanical energy demands during extrusion were the key drivers of GWP100 and PMFP. Fossil fuel combustion for heating, reliance on fossil-based electricity, and process emissions (methane, CO_2_, VOCs) during plastic melting were significant sources.^90^ PMFP from plastic waste processing might come from incomplete combustion of fossil fuels during heat generation and mechanical abrasion volatilization during extrusion and molding, and emissions from preprocessing stages and transportation, in cases where the waste is sourced from distant locations.^90^ We can minimize the impacts by (1) switching to renewable electricity for extrusion and heating, (2) better plastic pre-treatment to reduce emissions, and (3) using particulate filters and waste-heat recovery. Overall, higher plastic content correlated with greater impacts. Composition 1 (33.3% plastic) had the lowest impacts, while Composition 2 (53.3% plastic) had the highest matching trends observed in metal leaching. Finally, these findings indicate that the environmental performance of recycled plastic composites is governed primarily by the manufacturing processes rather than by the relatively small differences in material composition, highlighting the importance of improving processing efficiency and reducing energy demand during extrusion.

## 4 Conclusion

Plastic-sand-glass bricks produced from waste plastics in Timor-Leste demonstrated compressive strengths up to 24.3 ± 0.57 N/mm^2^, suitable for paving applications. Mechanical performance improved with lower plastic content, while UV weathering reduced strength, increased porosity, and considerably increased the leaching of inorganic contaminants, including Cr, Pb, Cu, amongst others which are metals of concern. Organic compounds, such as PAHs, were found below detection limits in both conditions (aged and unaged), while PFAS were mostly reduced after aging for all compositions except for Composition 4, which increased. Composition 1, with the lowest plastic fraction, combined the best mechanical strength, minimal leaching, and the most favorable life cycle impacts. In contrast, higher-plastic-content (e.g., Composition 2) performed poorly across durability, leaching, and environmental metrics.

Life-cycle assessment identified extrusion as the dominant contributor to climate change (GWP100) and particulate matter formation (PMFP), driven by fossil fuel-based energy demands and process emissions (CO_2_, methane, and VOCs). These impacts can be greatly reduced through renewable energy integration, waste heat recovery, process optimization, cleaner feedstocks, and particulate emission controls.

Overall, incorporating plastic waste with sand and glass is a useful waste management approach that allows for low-cost construction. However, formulation (for example, the quantity of plastic used) and processing choices (especially extrusion energy and emission controls) are some of the important operational factors that influence the relationship among durability, contaminant release, and environmental footprint. This study emphasizes the importance of optimizing material ratios and processing conditions while addressing pollutant release during the manufacture and use of blocks. Future work should focus on (1) effects of other environmental factors (rainfall, humidity, freeze–thaw) on durability and emissions; (2) occupational and environmental risks from manufacturing process emissions (such as particulates and volatile organic compounds), and evaluate effective controls aligned with LCA hotspots; (3) mechanisms of PFAS degradation and release under weathering; and (4) the persistence and ecological impacts of leached elements and compounds.

## Supplementary Material



## Data Availability

Data will be made available upon request.

## Notes

Photo of brick provided by Caltech Timor with measurements (Figure S1); preparation of the brick samples for further analysis (S2); elemental and chemical characterization of the samples (S3); compressive strength testing setup (Figure S2); analysis of inorganic elements (S5); leachate sample analysis for polycyclic aromatic hydrocarbons (S6); instrumental analysis of PFAS (S7); list of the 56 PFAS compounds included in the analysis (Table S1); analysis of non-targeted compounds (S9); impact categories, abbreviations, and units (Table S2); selected Ecoinvent activities (Table S3); elemental compositions of the different brick compositions results (Table S4); FTIR spectra and list of peaks and bond descriptions for the different materials present in the block compositions (Figure S3 and Table S5); XRM images of the internal structure of the blocks before and after UV exposure (Figures S4 and S5); pore volume distribution in blocks before and after UV exposure (Figure S7); example of 2D and 3D views of segmented pores in a block sample (Figure S6); concentrations of PAHs leached out from unexposed and UV-exposed block samples (Table S6); concentrations of PFAS leached out from unexposed and UV-exposed block samples (Table S7); correlation between metals and PFAS leaching vs material composition (variables) for unaged samples (Figure S8); correlation between material composition variables and PFAS and metal concentrations in aged and unaged samples (Figure S9); identification of plastic additives by non-targeted screening in leachate samples (Table S8); and the most contributing processes for the three top-contributing activities for each impact category of the LCA (Tables S9–S13) (PDF)

## #

Technische Universität Braunschweig, Institute of Infrastructure and Real Estate Management, Universitätsplatz 2, Braunschweig, 38106, Niedersachsen, Germany

## ∇

CSIR-Central Building Research Institute, Polymers, Plastics and Composites Division, CBRI Colony, Roorkee 247667, Uttarakhand, INDIA

## References

[1] Kumar R., Verma A., Shome A. (2021). Impacts of Plastic Pollution
on Ecosystem Services, Sustainable Development Goals, and Need to
Focus on Circular Economy and Policy Interventions. Sustainability.

[2] Moulai
Arbi Y., Mahmoudi N., Djebli A. (2023). Manufacturing and testing of waste
PET reinforced with sand bricks. J. Compos Mater..

[3] El-Metwally Y., Dewidar K., Ismail M., El-Mahallawi I. (2023). Optimization
of plastic waste integration in cement bricks. J. Eng. Appl. Sci..

[4] Małek M., Grzelak K., Łasica W. (2022). Cement-glass composite
bricks (CGCB) with interior 3D printed PET-G scaffolding. J. Build. Eng..

[5] Koppula N. K., Schuster J., Shaik Y. P. (2023). Fabrication and Experimental Analysis
of Bricks Using Recycled Plastics and Bitumen. J. Compos. Sci..

[6] Murat B. I. S., Kamalruzaman M. S., Azman M. H. N., Misroh M. F. (2020). Assessment
of Mechanical Properties of Recycled HDPE and LDPE Plastic Wastes. IOP Conf Ser. Mater. Sci. Eng..

[7] Estil, K. From Waste to Housing: Using Plastic Waste to Build Sustainable Housing in Haiti. In Florida Atlantic University ProQuest Dissertations & Theses; ProQuest, 2019.

[8] Musthofa M. M., Damayanti R. (2022). PErceptual
Study Of Plastic Waste Bricks For Sharia
Apartment In Indonesia. Adv. Civil Eng. Sustainable
Architect..

[9] Wangeci, M. Kenyatta university department of architecture & interior design arc 602: architectural research project building from plastic waste: opportunities and limitations in kenya; Kenyatta university, 2018.

[10] Ikechukwu A. F., Shabangu C. (2021). Strength and
durability performance of masonry bricks
produced with crushed glass and melted PET plastics. Case Stud. Constr. Mater..

[11] Kulkarni P., Ravekar V., Rama Rao P., Waigokar S., Hingankar S. (2022). Recycling
of waste HDPE and PP plastic in preparation of plastic brick and its
mechanical properties. Cleaner Mater..

[12] Tufa M., Tafesse D., Tolosa S., Murgan S. (2021). Study of sand-plastic
composite using optimal mixture design of experiments for best compressive
strength. Mater. Today Proc..

[13] Hamzah A. F., Alkhafaj R. M. (2022). An investigation
of manufacturing technique and characterization
of low-density polyethylene waste base bricks. Mater. Today Proc..

[14] Tulashie S. K., Dodoo D., Ibrahim A. A. W. (2022). Recycling plastic wastes
for production of sustainable and decorative plastic pavement bricks. Innov. Infrastruct. Solut..

[15] Webb H., Arnott J., Crawford R., Ivanova E. (2013). Plastic Degradation
and Its Environmental Implications with Special Reference to Poly­(ethylene
terephthalate). Polymers.

[16] Pickett, J. E. Weathering of Plastics. In Handbook of Environmental Degradation of Materials; Elsevier, 2018; pp. 163–184. DOI: 10.1016/B978-0-323-52472-8.00008-3

[17] Nguyen V. D., Hao J., Wang W. (2018). Ultraviolet
Weathering Performance of High-Density
Polyethylene/Wood-Flour Composites with a Basalt-Fiber-Included Shell. Polymers.

[18] Gewert B., MacLeod M., Breitholtz M. (2021). Variability in Toxicity of Plastic
Leachates as a Function of Weathering and Polymer Type: A Screening
Study with the Copepod *Nitocra spinipes*. Biol. Bull..

[19] Edike U. E., Ameh O. J., Dada M. O. (2023). Performance of polymer bricks produced
with plastic waste. Innov. Infrastruct. Solut..

[20] Qin Y., Liu Y., Wang J., Lu Y., Xu Z. (2022). Emission of PAHs, PCBs,
PBDEs and heavy metals in air, water and soil around a waste plastic
recycling factory in an industrial park, Eastern China. Chemosphere.

[21] Tang Z., Zhang L., Huang Q. (2015). Contamination and risk
of heavy metals in soils and sediments from a typical plastic waste
recycling area in North China. Ecotoxicol. Environ.
Saf..

[22] Yamashita, K. ; Kumagai, K. ; Noguchi, M. VOC Emissions from waste plastics during melting processes; Tohoku University Press, 2007.

[23] Amjad M. S., Diaz-Elsayed N. (2024). Evaluating the environmental impacts of brick production
from waste plastic. Manuf. Lett..

[24] Sahani K., Joshi B. R., Khatri K., Magar A. T., Chapagain S., Karmacharya N. (2022). Mechanical
Properties of Plastic Sand Brick Containing
Plastic Waste. Adv. Civil Eng..

[25] Premathilaka K. K. W., Liyanapathirana D. S., Leo C. J., Hu P. (2024). Application
of recycled waste glass to replace traditional quarried aggregates:
A comprehensive review. J. Build. Eng..

[26] Siddika A., Hajimohammadi A., Md M., Alyousef R., Ferdous W. (2021). Waste Glass
in Cement and Geopolymer Concretes: A Review on Durability and Challenges. Polymers.

[27] Mohajerani A., Vajna J., Cheung T. H. H., Kurmus H., Arulrajah A., Horpibulsuk S. (2017). Practical
recycling applications of crushed waste glass
in construction materials: A review. Constr.
Build. Mater..

[28] Lv Y., Huang Y., Yang J. (2015). Outdoor and accelerated
laboratory weathering of polypropylene: A comparison and correlation
study. Polym. Degrad. Stab..

[29] Bochen J., Ślusarek J. (2020). Analysis of
resistance to accelerated aging of the
top layer of small-sized concrete elements. A case study. Constr. Build. Mater..

[30] ASTM C 109/C 109M -08 Standard Test Method For Compressive Strength Of Hydraulic Cement Mortars (Using 2-In. Or [50-Mm] Cube Specimens)1; ASTM International, 2008.

[31] U.S. Environmental Protection Agency The Toxicity Characteristic Leaching Procedure Epa Method 1311; EPA, 1992

[32] Ling T. C., Poon C. S. (2014). Use of recycled CRT funnel glass as fine aggregate
in dry-mixed concrete paving blocks. J. Clean.
Prod..

[33] Liu Y., Zhuge Y., Chow C. W. K. (2020). Utilization of drinking
water treatment sludge in concrete paving blocks: Microstructural
analysis, durability and leaching properties. J. Environ. Manage..

[34] Zelinkova Z., Wenzl T. (2015). The Occurrence of 16 EPA PAHs in Food – A Review. Polycyclic Aromat. Compd..

[35] Cheng X., Forsythe J., Peterkin E. (2013). Some factors
affecting SPME analysis
and PAHs in Philadelphia’s urban waterways. Water Res..

[36] Peter K. T., Tian Z., Wu C. (2018). Using High-Resolution
Mass Spectrometry to Identify Organic Contaminants Linked to Urban
Stormwater Mortality Syndrome in Coho Salmon. Environ. Sci. Technol..

[37] Crawford, R. Life Cycle Assessment in the Built Environment; Routledge, 2011. DOI: 10.4324/9780203868171

[38] Novak, P. Timor-Leste’s Uncertain Future; Lowy Institute, 2023.

[39] Palmer J., Herat S. (2021). Ecotoxicity of Microplastic
Pollutants to Marine Organisms: a Systematic
Review. Water, Air, Soil Pollut..

[40] Cook E., Derks M., Velis C. A. (2023). Plastic
waste reprocessing for circular
economy: A systematic scoping review of risks to occupational and
public health from legacy substances and extrusion. Sci. Total Environ..

[41] Saha B., Eliason K., Golui D., Masud J., Bezbaruah A. N., Iskander S. M. (2023). Rare earth elements in sands collected from Southern
California sea beaches. Chemosphere.

[42] Hahladakis J. N., Velis C. A., Weber R., Iacovidou E., Purnell P. (2018). An overview of chemical additives
present in plastics:
Migration, release, fate and environmental impact during their use,
disposal and recycling. J. Hazard. Mater..

[43] Turner A., Filella M. (2021). Hazardous metal additives
in plastics and their environmental
impacts. Environ. Int..

[44] Iftikhar B., Alih S. C., Vafaei M. (2023). Experimental
study on
the eco-friendly plastic-sand paver blocks by utilising plastic waste
and basalt fibers. Heliyon.

[45] Aneke F. I., Shabangu C. (2021). Green-efficient masonry
bricks produced from scrap
plastic waste and foundry sand. Case Stud. Constr.
Mater..

[46] Hernandez L. M., Grant J., Fard P. S., Farner J. M., Tufenkji N. (2023). Analysis of
ultraviolet and thermal degradations of four common microplastics
and evidence of nanoparticle release. J. Hazardous
Mater. Lett..

[47] Fernlund G., Wells J., Fahrang L., Kay J., Poursartip A. (2016). Causes and
remedies for porosity in composite manufacturing. IOP Conf Ser. Mater. Sci. Eng..

[48] Gallos A., Paës G., Allais F., Beaugrand J. (2017). Lignocellulosic
fibers: a critical review of the extrusion process for enhancement
of the properties of natural fiber composites. RSC Adv..

[49] Chen J., Yu Z., Jin H. (2022). Nondestructive testing and evaluation techniques of
defects in fiber-reinforced polymer composites: A review. Front. Mater..

[50] Doğan M. (2021). Ultraviolet
light accelerates the degradation of polyethylene plastics. Microsc. Res. Technol..

[51] Adsul N., Kang S. T. (2024). Investigation of
the Compressive Strength and Void
Analysis of Cement Pastes with Superabsorbent Polymer. Polymers.

[52] Mehdikhani M., Gorbatikh L., Verpoest I., Lomov S. V. (2019). Voids in fiber-reinforced
polymer composites: A review on their formation, characteristics,
and effects on mechanical performance. J. Compos
Mater..

[53] Rodriguez A. K., Mansoor B., Ayoub G., Colin X., Benzerga A. A. (2020). Effect
of UV-aging on the mechanical and fracture behavior of low density
polyethylene. Polym. Degrad. Stab..

[54] Hamada H. M., Al-Attar A., Abed F. (2024). Enhancing sustainability
in concrete construction: A comprehensive review of plastic waste
as an aggregate material. Sustainable Mater.
Technol..

[55] Wen B., Wang H., Gao G., Zhang L., Yu Z., Wang Z. (2025). The Synergistic Utilization
of Glass Aggregates and Glass Powder
on the Thermal and Mechanical Properties of Concrete. Materials.

[56] Sajeevan M., Ahilash N., Shobijan J. (2025). Statistical investigation
of aggregate size and shape impact on porosity and compressive strength
of pervious concrete. Road Mater. Pavement Design.

[57] Tan C. J., Andriyana A., Ang B. C., Wong D. (2020). Mechanical deformation
and fracture mechanisms of polymeric fibres from the perspective of
fractography – A review. Eur. Polym.
J..

[58] Guo Y., Zhu S., Chen Y., Li D. (2019). Analysis and Identification of the
Mechanism of Damage and Fracture of High-Filled Wood Fiber/Recycled
High-Density Polyethylene Composites. Polymers.

[59] Hashemi R., Spring D. W., Paulino G. H. (2015). On small
deformation interfacial
debonding in composite materials containing multi-coated particles. J. Compos Mater..

[60] Mekideche S., Rokbi M., Rahmouni Z. E. A., Phiri R., Mavinkere
Rangappa S., Siengchin S. (2025). Manufacture and characterization
of lightweight sand-plastic composites made of plastic waste and sand:
Effect of sand types. Int. J. Lightweight Mater.
Manufact..

[61] Yue Y., Zhang J., Sun F. (2019). Heavy metal leaching
and distribution in glass products from the co-melting treatment of
electroplating sludge and MSWI fly ash. J. Environ.
Manage..

[62] Shan C., Pandyaswargo A. H., Onoda H. (2023). Environmental Impact of Plastic Recycling
in Terms of Energy Consumption: A Comparison of Japan’s Mechanical
and Chemical Recycling Technologies. Energies.

[63] Jansen M. A. K., Andrady A. L., Bornman J. F. (2024). Plastics in the environment
in the context of UV radiation, climate change and the Montreal Protocol:
UNEP Environmental Effects Assessment Panel, Update 2023. Photochem. Photobiol. Sci..

[64] Conesa J. A., Nuñez S. S., Ortuño N., Moltó J. (2021). PAH and POP
Presence in Plastic Waste and Recyclates: State of the Art. Energies.

[65] Alassali A., Calmano W., Gidarakos E., Kuchta K. (2020). The degree and source
of plastic recyclates contamination with polycyclic aromatic hydrocarbons. RSC Adv..

[66] Martín J. C., Moltó J., Ortuño N., Fullana A., Conesa J. A. (2025). Assessment
of toxic polycyclic aromatic hydrocarbons (PAH) in recycled plastics:
A comparative study of LDPE, HDPE, PET, and PP. Res., Conservat. Recycl. Adv..

[67] Bečanová J., Melymuk L., Vojta Š., Komprdová K., Klánová J. (2016). Screening for perfluoroalkyl acids
in consumer products, building materials and wastes. Chemosphere.

[68] Llorca M., Farré M., Karapanagioti H. K., Barceló D. (2014). Levels and
fate of perfluoroalkyl substances in beached plastic pellets and sediments
collected from Greece. Mar. Pollut. Bull..

[69] Weis J. S., Alava J. J. (2023). (Micro)­Plastics
Are Toxic Pollutants. Toxics.

[70] Islam S., Kekre K. M., Shah T. A., Tsai P. -C., Ponnusamy V. K., Andaluri G. (2025). Unraveling the complexities
of microplastics and PFAS
synergy to foster sustainable environmental remediation and ecosystem
protection: A critical review with novel insights. J. Hazard. Mater. Adv..

[71] Hellsing M. S., Josefsson S., Hughes A. V., Ahrens L. (2016). Sorption of perfluoroalkyl
substances to two types of minerals. Chemosphere.

[72] Zenobio J. E., Salawu O. A., Han Z., Adeleye A. S. (2022). Adsorption of per-
and polyfluoroalkyl substances (PFAS) to containers. J. Hazard. Mater. Adv..

[73] Shi Y., Almuhtaram H., Andrews R. C. (2023). Adsorption of Per- and Polyfluoroalkyl
Substances (PFAS) and Microcystins by Virgin and Weathered Microplastics
in Freshwater Matrices. Polymers.

[74] Zimmermann L., Bartosova Z., Braun K., Oehlmann J., Völker C., Wagner M. (2021). Plastic Products Leach Chemicals That Induce *In Vitro* Toxicity under Realistic Use Conditions. Environ. Sci. Technol..

[75] Marques C., Hadjab F., Porcello A. (2024). Mechanistic Insights
into the Multiple Functions of Niacinamide: Therapeutic Implications
and Cosmeceutical Applications in Functional Skincare Products. Antioxidants.

[76] Fiume M. M., Heldreth B. A., Bergfeld W. F., Belsito D.V., Hill R. A., Klaassen C. D., Liebler D. C., Marks J. G., Shank R. C., Slaga T. J. (2015). Safety Assessment of
Ethanolamides as Used in Cosmetics. Int J Toxicol..

[77] Akoueson F., Chbib C., Monchy S. (2021). Identification and quantification
of plastic additives using pyrolysis-GC/MS: A review. Sci. Total Environ..

[78] Su Y., Li F., Xiao X., Li H., Wang D., You J. (2023). Ecological
risk of galaxolide and its transformation product galaxolidone: evidence
from the literature and a case study in Guangzhou waterways. Environ. Sci.: Processes Impacts.

[79] Bolgar, M. ; Hubball, J. ; Groeger, J. ; Meronek, S. Handbook for the Chemical Analysis of Plastic and Polymer Additives; CRC Press, 2015. DOI: 10.1201/b19124

[80] Rohrich R. J., Brown S., Brown T., Taub P. J. (2025). Role of tranexamic
acid (TXA) in plastic and reconstructive surgery: A national perspective. J. Plastic, Reconstruct. Aesthetic Surgery.

[81] Wang Y., Chen S., Du K. (2021). Traditional herbal medicine:
Therapeutic potential in rheumatoid arthritis. J. Ethnopharmacol..

[82] Silva D. J. D., Wiebeck H. (2022). ATR-FTIR Spectroscopy Combined with Chemometric Methods
for the Classification of Polyethylene Residues Containing Different
Contaminants. J. Polym. Environ..

[83] Fauser P., Vorkamp K., Strand J. (2022). Residual additives
in marine microplastics
and their risk assessment – A critical review. Mar. Pollut. Bull..

[84] DeGennaro M. (2015). The mysterious
multi-modal repellency of DEET. Fly.

[85] Kilinc M., Cakal G. O., Bayram G., Eroglu I., Özkar S. (2015). Flame retardancy
and mechanical properties of pet-based composites containing phosphorus
and boron-based additives. J. Appl. Polym. Sci..

[86] Soni M. G., Burdock G. A., Taylor S. L., Greenberg N. A. (2001). Safety
assessment of propyl paraben: a review of the published literature. Food Chem. Toxicol..

[87] Goyal H., Kumar R., Mondal P. (2023). Life cycle
analysis of paver block
production using waste plastics: Comparative assessment with concrete
paver blocks. J. Clean. Prod..

[88] Budihardjo M. A., Iqbal M. J., Puspita A. S., Ramadan B. S., Chegenizadeh A., Nugroho A. T. D. (2026). Sustainability
and performance of recycled polyethylene
terephthalate paving blocks: a life cycle assessment. J. Mater. Cycles Waste Manage..

[89] Tushar Q., Salehi S., Santos J. (2023). Application
of recycled
crushed glass in road pavements and pipeline bedding: An integrated
environmental evaluation using LCA. Sci. Total
Environ..

[90] Adekanmbi A. O., Ani E. C., Abatan A., Izuka U., Ninduwezuor-Ehiobu N., Obaigbena A. (2024). Assessing
the environmental and health impacts of plastic
production and recycling. World J. Biol. Pharmacy
Health Sci.

