# Supplementary material for: Environmental Impacts and Life Cycle Assessment of Recycled Plastic Bricks
Source: ACS Omega. 2026 Sep 8;11(37):55596–610. doi: 10.1021/acsomega.6c04677 (PMC13613908; doi:10.1021/acsomega.6c04677)
Supplement: Supplementary file 1 [file ao6c04677_si_001.pdf]

Supplementary Information for
Environmental Impacts and Life Cycle Assessment of Recycled Plastic Bricks

Sirri Neba Nforsoh[a], Kayla Kurtz[a], Marlene Prien[a,ǂ], Jitka Becanova[b], Simon Vojta[b], Rui Wang[c], Rakesh Paswan[a,¥], Karla Pozo[de], Rainer Lohmann[b], Bradley Clarke[c], Sumanta Das[a], Vinka Oyanedel-Craver[a]*.

[a]Department of Civil and Environmental Engineering, University of Rhode Island, 2 East Alumni Avenue, Kingston, RI, 02881, United States
[b]University of Rhode Island, Graduate School of Oceanography, 215 South Ferry Road, Narragansett, Rhode Island, 02882, United States
[c]Australian Laboratory for Emerging Contaminants (ALEC), School of Chemistry, The University of Melbourne, Grattan Street, Melbourne, Victoria 3010, Australia
[d]Universidad San Sebastián, Facultad de Ingeniería, Arquitectura y Diseño, Lientur 1457, Concepción, 4080871, Bio-Bio, Chile
[e]Masaryk University, Faculty of Science, RECETOX, Kamenice 753/5, Brno, 625 00, Czech Republic

[ǂ]Current address: Technische Universität Braunschweig, Institute of Infrastructure and Real Estate Management, , Universitätsplatz 2, Braunschweig, 38106, Niedersachsen, Germany
[¥]Current address: CSIR-Central Building Research Institute, Polymers Plastics and composites Division, CBRI Colony, Roorkee, 247667, Uttarakhand, INDIA

*Corresponding Author
Vinka Oyanedel-Craver.
Email: craver@uri.edu
Department of Civil and Environmental Engineering, University of Rhode Island, 2 East Alumni Avenue, Kingston, RI, 02881, United States

S1: A photo of a sample of the brick from Timor-Leste
Dimensions: (l x w x h) of 0.20m x 0.17m x 0.05m.

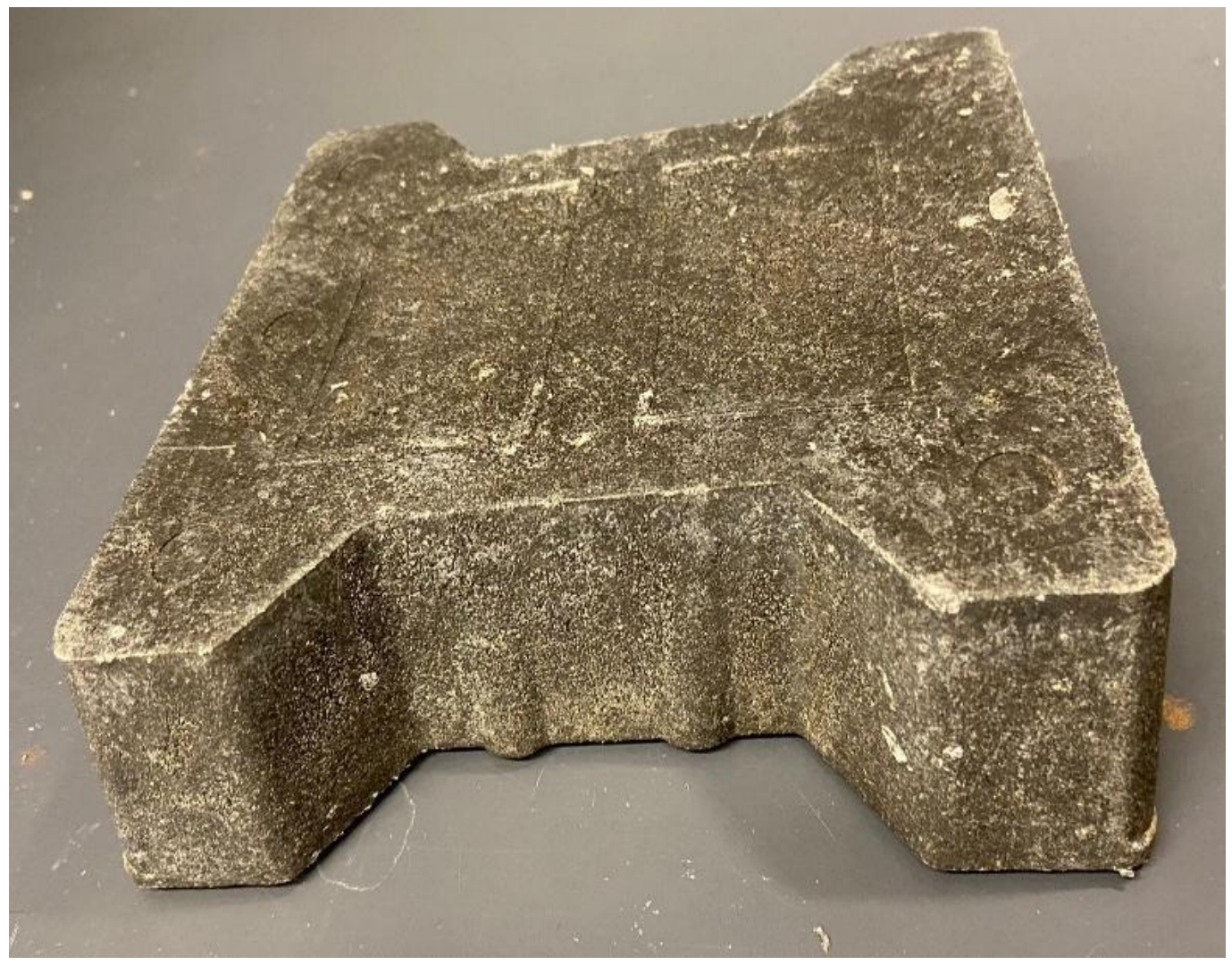

Figure S1. Photo of brick provided by Caltech Timor with measurements.

S2: Preparation of the brick samples for further analysis
Bricks were shipped to the University of Rhode Island for testing. Samples were cut with a table saw into 1 × 1 × 1 in. cubes for 3D imaging and 2 × 2 × 2 in. cubes for compressive strength testing. After strength testing, blocks were pulverized using a W41062 12-Ton Hydraulic Shop Press (Guangduan, China) and sieved to 10–15 mm for elemental and chemical composition analysis, and <10 mm for leachate analysis. To account for material heterogeneity, representative subsamples of equal mass were collected from all sides of each brick.

S3: Elemental and chemical characterization of the samples
Elemental composition of the four brick formulations was analyzed using an Energy Dispersive X-ray Fluorescence Spectrometer (EDXRF; Shimadzu EDX-8100, Shimadzu Corporation, Japan). Samples (10-15 mm) were placed in 31 mm open-ended X-cell EDX type 3571 vials and analyzed in triplicate for each composition. Measurements were performed using PCEDX Navi and Pro software under quantitative analysis settings, with a 10 mm collimator in air atmosphere.
Chemical composition was further characterized using attenuated total reflectance Fourier-transform infrared spectroscopy (ATR-FTIR; Shimadzu IRTracer-100, Shimadzu Corporation, Japan) with LabSolutions IR software. Analyses were conducted in absorbance mode with 32 scans over a spectral range of 400-4000 cm^{-1}. The detector operated in standard mode with the

internal beam option, without liquid nitrogen cooling. Triplicate measurements were performed for each composition using 10-15 mm samples.

S4: Compressive strength testing setup

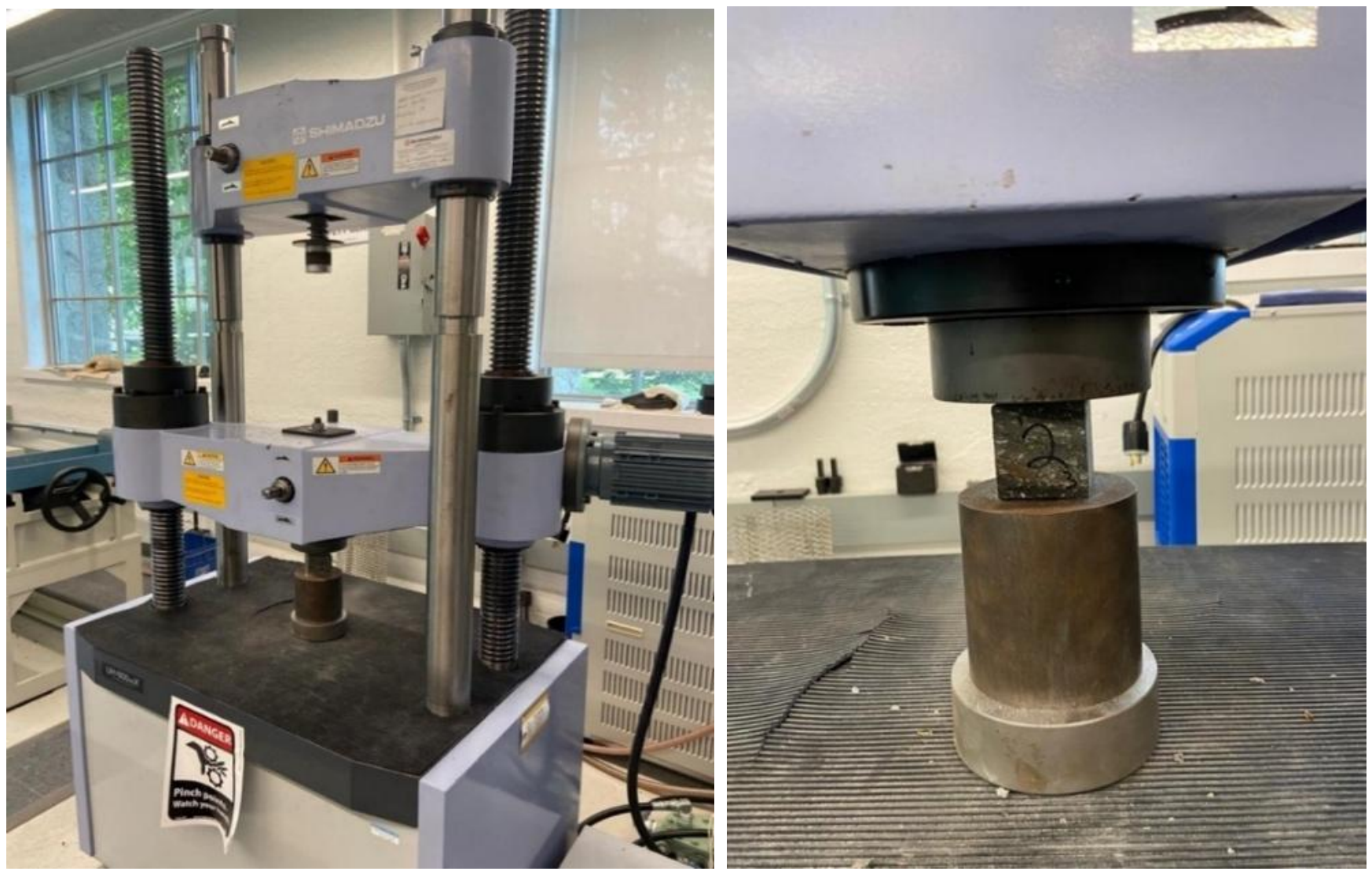

Figure S2. UH-600 kNX Universal Testing Compression Machine used for compressive strength testing of the four brick compositions.

S5: Analysis of inorganic elements

The inclusion of these eight metals is justified by using some in plastic manufacturing as additives, such as zinc oxide, which serves as stabilizers[1], and their potential environmental hazards, particularly when present in specific quantities, also influenced the choice of these elements [2]. Preceding the analytical procedure, the samples underwent filtration utilizing a 0.45um filter and were subsequently transferred into 10ml sterile polypropylene tubes. Filtered leachate samples were then analyzed in triplicate, controlled by the Lab solutions software. Standards were prepared using the selected metals stock standard solutions of 1000ug/mL concentration purchased from Fisher chemical. And diluted to respective concentrations.

S6: Leachate sample analysis for polycyclic aromatic hydrocarbons

Separation was accomplished using a 30 m x 280 um x 0.25 um Restek RTX-5 MS capillary column (Restek Corp., Bellefonte, PA, USA). The GC inlet temperature was set to 280°C, and it was run in spitless mode. The MS was set to select ion monitoring (SIM). The column was initially kept at 60 °C for 5 minutes before being raised to 150 °C at a rate of 30 °C/min, then to 200 °C and 250 °C at rates of 4 °C/min and 6 °C/min, respectively. Finally, it was raised to 320 °C at a rate of 15 °C/min and held there for 10 minutes to clean [3]. Mixed stock solution of 16 PAH standards (2000ppb) was purchased from Fisher scientific, USA. Acetonitrile, >99.9% (Muskegon, MI, USA) and 99.9% methanol HPLC grade was acquired from Fisher scientific (Fair

Lawn, NJ, USA). Concentrations of the calibration standards were between 0.0 and 3.0ug/L. For each of the four compositions and the control, samples were analyzed in duplicate. Solid phase micro-extraction (SPME) separation of leachate samples was carried out using polydimethylsiloxane (PDMS) coated fibers (Shimadzu Corporation, MA, USA). The incubation temperature was set at 60 ^{O}C. The agitation speed was 250 rpm, the extraction time was 30 minutes, and the desorption time was 5 minutes. For maximum cleaning, the fiber was conditioned for 30 minutes at 270 ^{O}C under helium flow and then cleaned twice at the start of each sample set by running a fiber blank [3].

S7: Instrumental analysis of PFAS

A Phenomenex Gemini 3um C18 110Å 50x2mm LC analytical column preceded with a Phenomenex SecurityGuard cartridge was used for the analyte separation. Another Phenomenex Gemini 5um C18 110 Å 50x4.6mm LC analytical column was used to delay the PFASs instrumental contribution. The aqueous mobile phase (MPA) was 10mM ammonium acetate in water and the organic mobile phase (MPB) was 10mM ammonium acetate in methanol. LC parameters were set as follows: flow 0.3mL/min, injection 20uL, column oven 45°C. Solvent gradient of MPB gradually increased from 40% to 80% (1 to 5.5 min), 80% to 100% (5.5 to 7 min), then hold for one minute and finally drop to 40% (8 to 8.5 min) and hold for another 6.5 minutes.

For the quantification of the target analytes, a HRMSMS (MRM HR) method was used. The negative ESI with the following parameters was used: curtain Gas at 30 psi, ion source gas 1 at 40 psi, ion source gas 2 at 60 psi, temperature 450°C.

S8: Targeted PFAS compounds and abbreviations included in the analysis

Table S1: List of the 56 PFAS compounds included in the analysis

**Acronym**	**Compound Name**
PFPrA	Perfluoropropanoic acid
PFBA	Perfluorobutanoic acid
PFPeA	Perfluoropentanoic acid
PFHxA	Perfluorohexanoic acid
PFHpA	Perfluoroheptanoic acid
PFOA	Perfluorooctanoic acid
PFNA	Perfluorononanoic acid
PFDA	Perfluorodecanoic acid
PFUdA	Perfluoroundecanoic acid
PFDoA	Perfluorododecanoic acid
PFTrDA	Perfluorotridecanoic acid
PFTeDA	Perfluorotetradecanoic acid
3:3 FTCA (FPrPA)	2H,2H,3H,3H-Perfluorohexanoic acid
5:3 FTCA (FPePA)	2H,2H,3H,3H-Perfluorooctanoic acid
7:3 FTCA (FHpPA)	2H,2H,3H,3H-Perfluorodecanoic acid
8:2 FTCA (FOEA)	8:2 Perfluorocarboxylic acid

PFPrS	Perfluropropanesulfonic acid
PFBS	Perfluorobutanesulfonic acid
PFPeS	Perfluoropentanesulfonic acid
L-PFHxS	Perfluorohexanesulfonic acid
Br-PFHxS	Perfluorohexanesulfonic acid
PFHpS	Perfluroheptanesulfonic acid
L-PFOS	Perfluorooctanesulfonic acid
Br-PFOS	Perfluorooctanesulfonic acid
PFNS	Perfluorononanesulfonic acid
PFDS	Perfluorodecanesulfonic acid
MeFBSA	N-Methylperfluorobutane sulfonamide
N-EtFBSA-M	N-Ethylperfluorobutane sulfonamide
FBSA	Perfluorobutyl sulfonamide
FPeSA	Perfluoropentane sulfonamide
FHxSA	Perfluorohexane sulfonamide
FHpSA	Perfluoroheptane sulfonamide
FOSA	Perfluorooctane sulfonamide
L-N-MeFOSAA	N-methyl perfluorooctanesulfonamidoacetic acid
Br-N-MeFOSAA	N-methyl perfluorooctanesulfonamidoacetic acid
L-N-EtFOSAA	N-ethyl perfluorooctanesulfonamidoacetic acid
Br-N-EtFOSAA	N-ethyl perfluorooctanesulfonamidoacetic acid
MeFOSA	N-Methylperfluorooctane sulfonamide
EtFOSA	N-Ethylperfluorooctane sulfonamide
HFPO-DA (Gen X)	Hexafluoropropylene oxide dimer acid
DONA (ADONA)	4,8-Dioxa-3H-perfluorononanoic acid
9ClPF3ONS	9-Chlorohexadecafluoro-3-oxanonane-1-sulfonic acid
11ClPF3OUdS	11-Chloroeicosafluoro-3-oxaundecane-1-sulfonic acid
3,6-OPFHpA	Nonafluoro-3,6-dioxaheptanoic acid
PF4OPeA	Perfluoro-3-methoxypropanoic acid
PF5OHxA	Perfluoro-4-methoxybutanoic acid
PFECHS	Perfluoroethylcyclohexanesulfonic acid
PFEESA	Perfluoro(2-ethoxyethane)sulfonic acid
N-AP-FHxSA	N-[3-(Dimethylamino)propyl] Perfluorohexane sulfonamide
N-TAmP-FHxSA	N-[3-(Trimethylamino)propyl] Perfluorohexane sulfonamide
4:2 FTS	1H,1H,2H,2H-Perfluorohexanesulfonic acid
6:2 FTS	1H,1H,2H,2H-Perfluorooctanesulfonic acid
8:2 FTS	1H,1H,2H,2H-Perfluorodecanesulfonic acid
10:2 FTS	1H,1H,2H,2H-Perfluorododecanesulfonic acid

S9: Analysis of non-targeted compounds

SPE was done using a vacuum manifold (Sigma-Aldrich, MO, USA) with 500 mg hydrophilic-lipophilic balanced (HLB) cartridges (Waters Corporation, MA, USA). Cartridges were preconditioned with LC-MS grade methanol and rinsed with LC-MS grade water (Fisher

Scientific, NJ, USA). Each sample (200 mL) was spiked with 80 ng/mL PFAS 327v surrogate standard, eluted with 4 mL of 1% ammonium hydroxide in methanol, and concentrated to 1 mL under nitrogen. Extracts were wrapped in foil, shipped on ice to the University of Melbourne (4-day transit), and analyzed at the Australian Laboratory for Emerging Contaminants (ALEC). Elution was performed sequentially with 4 mL methanol/acetonitrile (1:1, 0.1% NH_4OH) and 4 mL acetonitrile. Samples were evaporated to 50 µL, reconstituted in 950 µL methanol, and prepared with internal standards (10 µL of 1 mg/L ISTD per 90 µL sample). Method and field blanks were processed identically for quality control.

Chromatographic separation used an Agilent ZORBAX Eclipse Plus C18 column (2.1 × 100 mm, 1.8 µm) with a matching guard cartridge (2.1 × 5 mm, 1.8 µm) at 45 °C. For ESI^+, the mobile phases were 5 mM ammonium acetate with 0.1% formic acid in water (aqueous) and 0.1% formic acid in methanol (organic); for $ESI^–$, the aqueous phase was 2 mM ammonium acetate in water and the organic phase was methanol. The gradient (0.4 mL $min^{–1}$; 1 µL injection) was 5% organic at 0–1 min, 50% at 4 min, 100% at 17–20 min, and back to 5% at 20.1–22.5 min. Mass spectra were acquired over m/z 50–1000 in data-independent acquisition (DIA; 30 eV; 5 Hz) and data-dependent acquisition (DDA; top-5 precursors; stepped 10/20/40 eV).

Raw files were converted to mzML using MSconvert[4] Data reduction and feature extraction were performed with the open-source Python package PyHRMS (https://pypi.org/project/pyhrms/), including feature detection/alignment (retention-time–exact-mass pairs), grouping of adducts ($[M+H]^+$, $[M+Na]^+$, $[M+NH_4]^+$, $[M+K]^+$) and isotopologues (C/Cl/S/Br), and association of MS^2 spectra from DIA/DDA. Features were retained if intensity exceeded 500 counts and peak area was ≥5-fold greater than blanks. Annotations were matched against (i) an in-house database (~200 chemicals) characterized by experimental RT, accurate mass, and MS^2 fragments, (ii) the NORMAN Suspect List Exchange[5], and (iii) MassBank of North America (LC–MS spectra)[6]. Identification confidence followed standard criteria: Level 1 required accurate mass (<5 ppm), RT (<0.1 min), and ≥2 fragment matches (mass error <0.015 Da) to reference standards; Level 2 required accurate mass plus ≥3 fragment matches to library spectra.

MS/MS data (m/z) were collected for structure identification utilizing data-dependent acquisition and lists of favored precursors derived from first MS-only screening. Nontarget feature extraction, adduct detection/grouping, and alignment were all done in MassHunter Profinder. Features (precise mass-retention time pairs) were loaded into Mass Profiler Professional for further filtering and analysis.

S10: List of the different impact categories

Table S2. Impact categories, abbreviations and units

Impact category	Abbreviations	Unit
Agricultural land occupation	ALOP	m^2 -year
Fossil depletion	FDP	Kg oil-eq

Freshwater eutrophication	FEP	Kg P-eq
Fresh water ecotoxicity	FETPinf	Kg 1.4-DCB-eq
Climate change	GWP100	Kg CO_2-eq
Human toxicity	HTPinf	Kg 1.4-DCB-eq
Ionization radiation	IRP_HE	Kg U235-eq
Metal depletion	MDP	Kg Fe-eq
Marine eutrophication	MEP	Kg N -eq
Marine ecotoxicity	METPinf	Kg 1.4-DCB-eq
Natural land transformation	NLTP	m^2
Ozone depletion	ODPinf	Kg 1.4-CFC-eq
Particulate matter formation	PMFP	Kg PM10 -eq
Photochemical oxidant formation	POFP	Kg NMVOC -eq
Terrestrial acidification	TAP100	kgSO_2 -eq
Terrestrial ecotoxicity	TETPinf	Kg 1.4-DCB-eq
Urban land Occupation	ULOP	m^2 -year
Water depletion	WDP	m^3 water-eq

S11: List of the different selected ecoinvent activities used in the LCA

Table S3. Selected Ecoinvent activities.

	Process	Ecoinvent Activity	Comments
A1: Raw Material Supply	Sieving and drying sand	Silica sand production (RoW)	Activity includes the mining of sand, the heat production required for drying the sand and ends with the final product 'silica sand'.
	Sorting, washing, and crushing waste glass	Treatment of waste glass from unsorted public collection, sorting (RoW)	Activity includes the collection and transportation of the by-product generated in the glass production and its sorting process. The final product is sorted glass cullets.

	Collecting waste plastic, removing any dirt, washing, drying and shredding waste plastic	Market for polyethylene terephthalate, granulate, amorphous, recycled (RoW)	This activity includes the collection, transportation, sorting, processing of waste PET and ends with recycled PET granulates. The same activity is used for HDPE and LDPE as there is no specific activity available for these two types of plastic.
	Drying the PET before it enters the extruder	Drying of maize straw and whole-plant (RoW)	The activity includes the energy demand for evaporating 1 kg of water when maize plants are dried at a temperature of 110-120°C. The infrastructure, i.e. machinery and building, needed for this process is also included.
	Mixing the raw materials – plastic granulate, sand and glass cullets	Plaster mixing (RoW)	This activity includes the electricity demand for mixing 1 kg of plaster.
	Melting and extruding the block mixture	Extrusion, plastic pipes (RoW)	This activity includes the electricity and heat required for the extrusion process, and the extrusion of 1 kg of plastic pipes. Extruding plastic pipes is the closest activity available to model the extrusion of the brick mixture.
	Mold release for the block molds	Market for vegetable oil, refined (GLO)	This activity includes three types of oil. Soybean oil and palm oil make up the largest share of the market, while cottonseed oil only accounts for around 6%. There is

			no activity available that represents a specific mold release. According to companies and literature oil can also be used.
	Energy demand of the hydraulic press used to compact the pavers. The compressive force applied is 15 MPa.	Electricity voltage transformation from high to medium voltage (ID)	This activity represents the production of 1 kWh electricity and includes the four sources biogas, natural gas, lignite and hard coal. The location of the activity is set in Indonesia and represents the mix of electricity sources as accurately as possible.
	Water to cool down the pavers	Tap water production, underground water without treatment (RoW)	This activity delivers tap water. The product can be used in various household applications, and for a wide variety of industrial uses like fabricating, processing, washing, diluting, cooling, etc.

S12: Elemental composition of bricks from XRF analysis

Table S4. Elemental compositions of the different brick compositions

**Composition**	**Elements Present**														
	Si	K	Ca	Al	Ti	Cr	Mn	Fe	Cu	Zn	Pb	Br	Sr	Ba	V
1	X	X	X	X	X	X	X	X	X	X	X	X	X		
2	X	X	X	X	X	X	X	X	X	X	X		X		X
3	X	X	X	X	X	X	X	X	X	X			X	X	X
4	X	X	X	X	X	X	X	X	X	X	X	X			

S13: FTIR spectra and list of peaks and bond descriptions for the different materials present in the block compositions from FTIR results

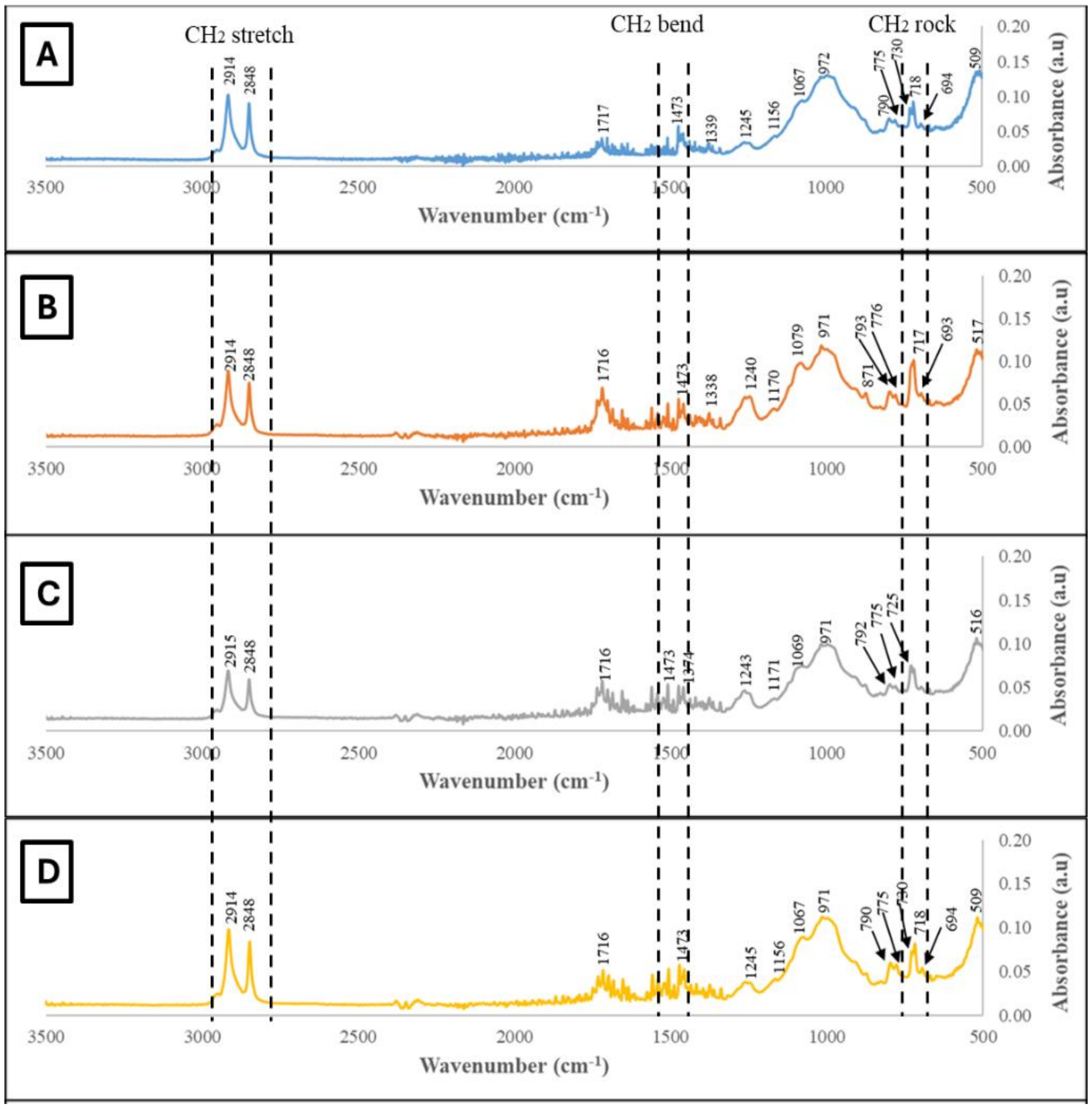

Figure S3. FTIR spectra for (A) composition 1 (glass, sand, PET and HDPE), (B) composition 2 (glass, sand, PET and HDPE), (C) composition 3 (glass, PET, HDPE and LDPE). and (D) composition 4 (sand, PET, HDPE and LDPE). Three replicate spectra were averaged for each composition, all replicates were similar, and no peaks were added or removed by averaging. PET and HDPE are present in all compositions. The arrows and highlighted peaks numbers are principal wavenumbers representing a chemical bond for different polymer types.

Table S5. Peaks and bond descriptions of the different materials present in the block compositions from FTIR results

Peak numbers/ bands	Bond/bond types	Material	References
509cm^{-1} to 517cm^{-1}	Si-O asymmetrical bending vibrations	Sand	[7,8,9]
693cm^{-1} to 694cm^{-1}	Si-O symmetrical bending vibrations	sand	[7–9]
775cm^{-1} to 776cm^{-1} and 790 cm^{-1} to 793 cm^{-1}	Si-O symmetrical stretching vibrations	sand	[7–9]
2914 cm^{-1}and 2915 cm^{-1}	CH2 asymmetric stretching	HDPE, LDPE, PET	[10–13]

2848cm^{-1}	CH2 symmetric stretching	HDPE, LDPE	[10,11,12]
1473 cm^{-1}	CH2 bending deformation	HDPE, LDPE	[10–12,14]
1374 cm^{-1} and 1375 cm^{-1}	Wagging deformation	HDPE, LDPE, PET	[10–12,14]
725 cm^{-1} to 730 cm^{-1} and 717 cm^{-1} to 718 cm^{-1}	CH2 rocking deformation	HDPE, LDPE	[10–13]
971 cm^{-1}	O–CH2 or C(=O)–O stretching	PET	[15]
1338 cm^{-1} to 1339 cm^{-1}	CH2 wagging	PET	[15]
1473 cm^{-1}	CH2 bending	PET	[15]
3000 to 2840 cm^{-1}	O–H bending and stretching vibration	Glass	[16]
1067 cm^{-1} to 1156 cm^{-1}	asymmetric stretching vibration network of Si–O–Si	Glass	[17]
750 cm^{-1} to 606 cm^{-1}	symmetric stretching vibration network	Glass	[16]

S14: XRM images of the internal structure of the blocks (Before UV exposure)

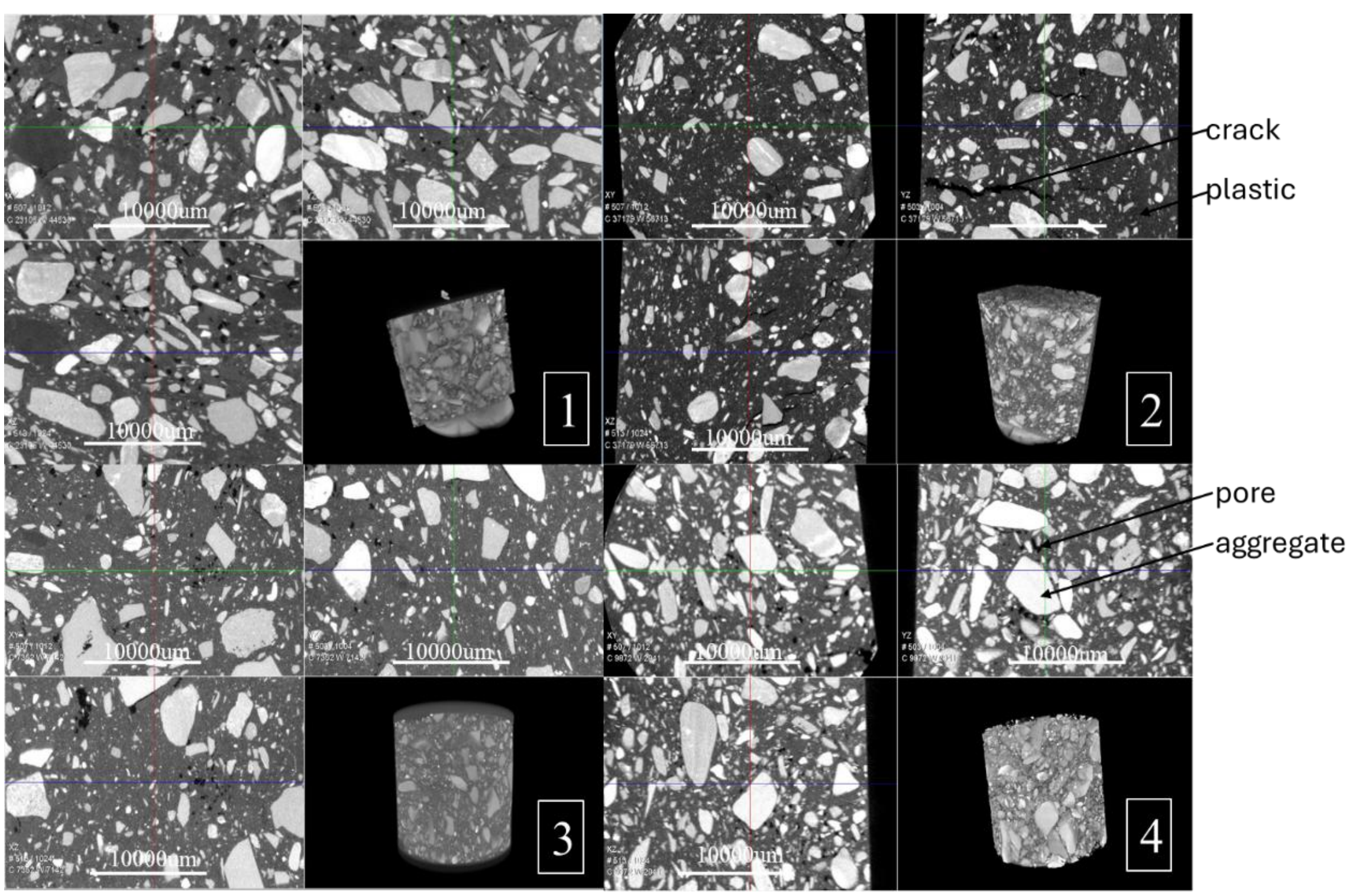

Figure S4. 3D XRM images of the unexposed samples for compositions 1, 2, 3 and 4. The figure presents the different axis of the blocks imaged and a 3D view of the block**.**

S15: XRM images of the internal structure of the blocks (After UV exposure)

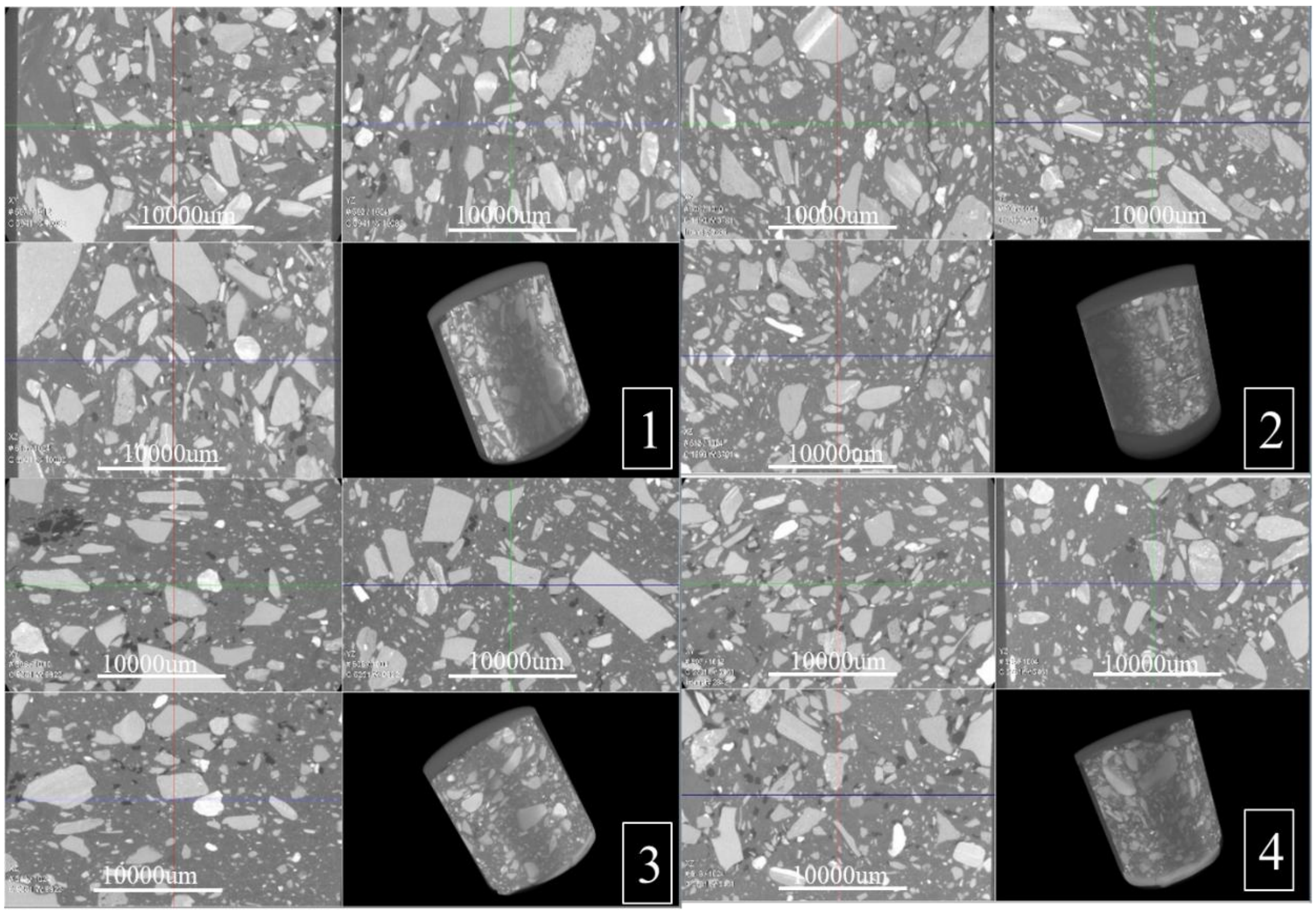

Figure S5. 3D XRM images of the UV exposed samples for compositions 1, 2, 3 and 4. The figure presents the different axis of the blocks imaged and a 3D view of the block**.**

S16: An example of 2D and 3D views of segmented pores in a block sample

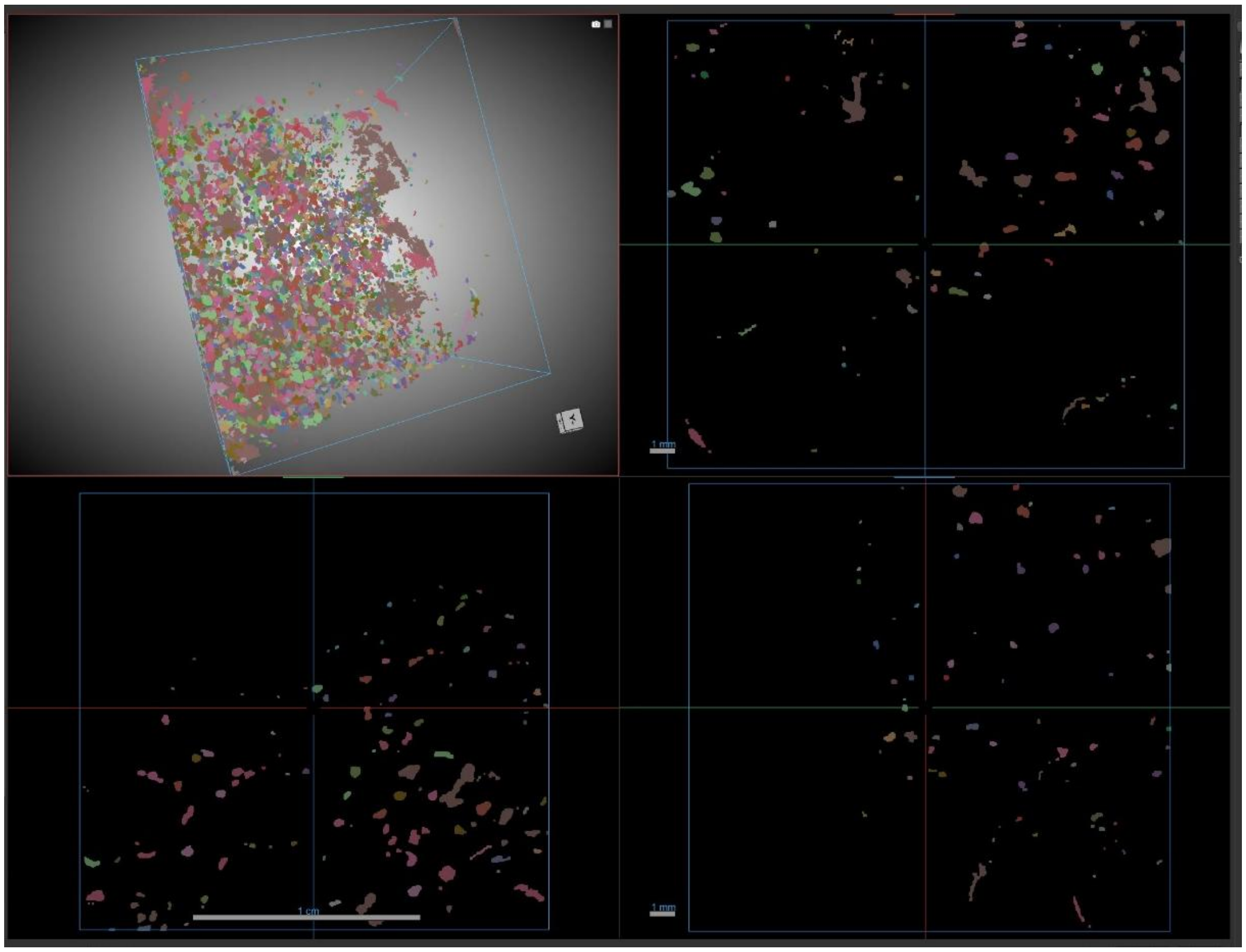

Figure S6. An example of a 2D and 3D views of segmented pore distributions a block sample volume

S17: Pore volume distribution in blocks before UV and after UV exposure

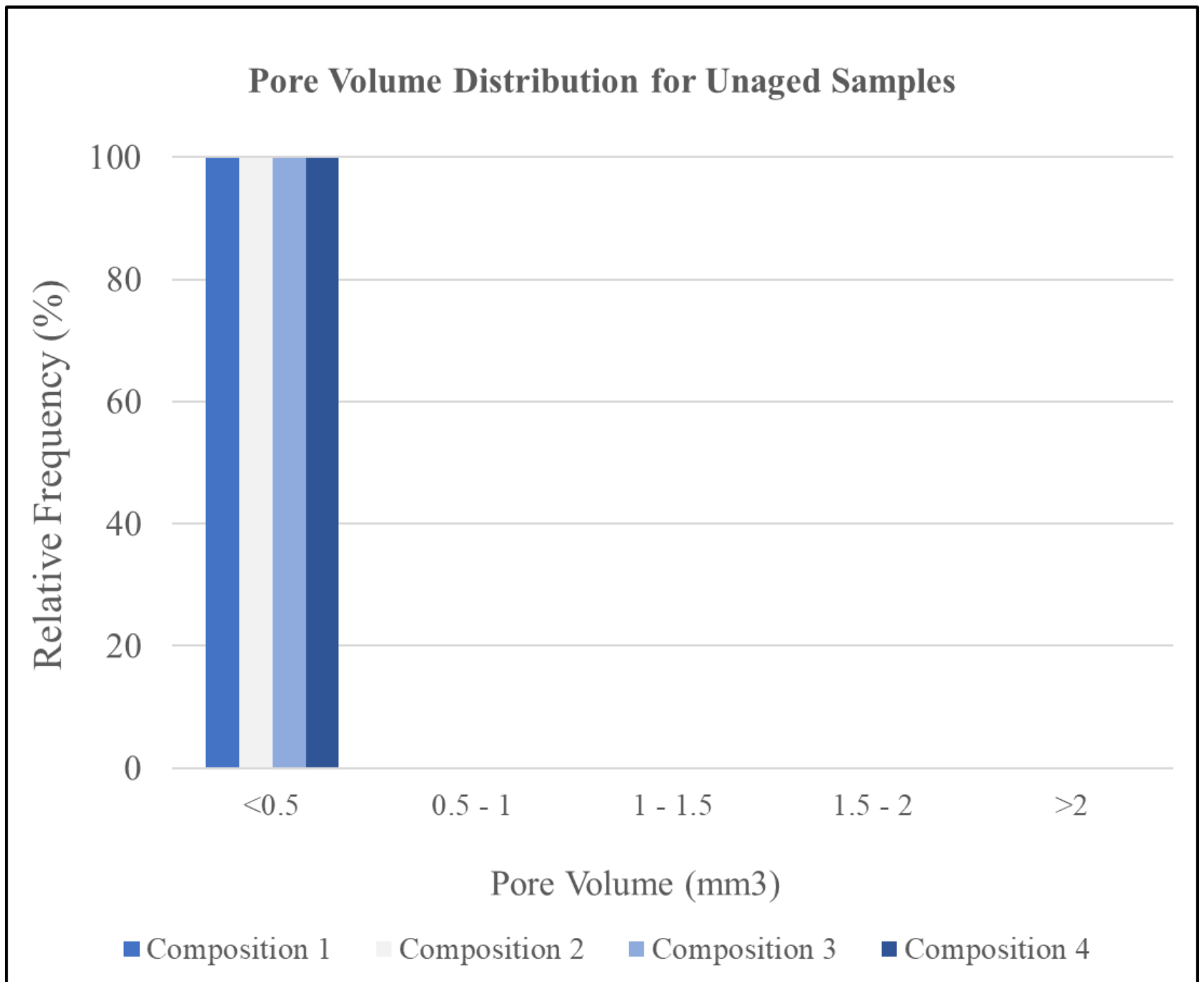

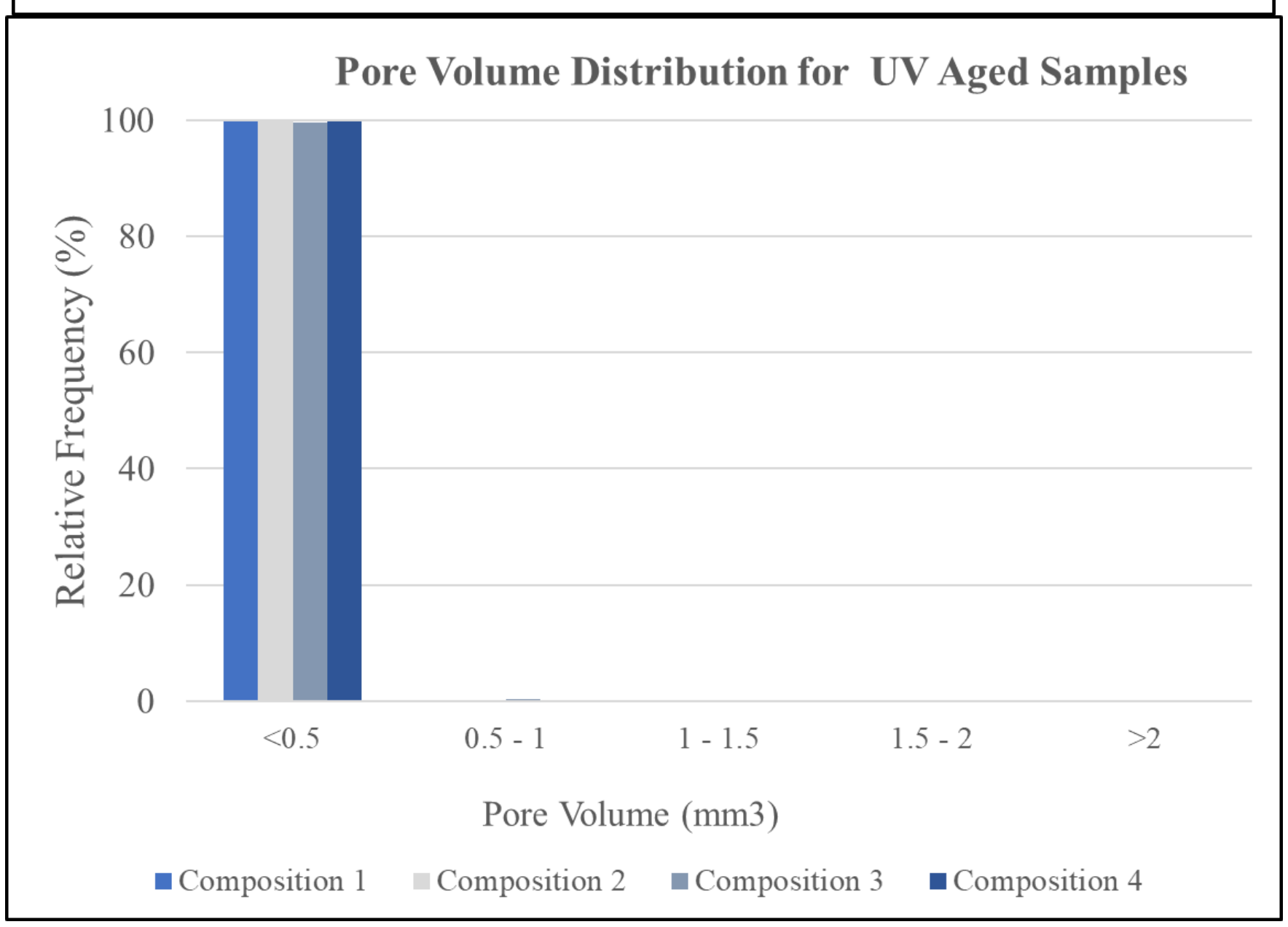

Figure S7. pore volume distribution in blocks before UV and after UV exposure

S18: PAHs leachate concentrations for the UV exposed and unexposed blocks

Table S6. Concentrations of PAHs leached out from unexposed and UV exposed block samples

PAH Compound	Concentrations (ug/l)									
	Before UV exposure				After UV exposure				Limits	
	C1	C2	C3	C4	C1	C2	C3	C4	LOD	LOQ
Napthalene	n.d	0.03	0.05	0.07	0.07	n.d	0.02	0.07	0.94	3.12

Acenaphthylene	n.d	n.d	n.d	n.d	n.d	n.d	n.d	0.04	0.58	1.94
Acenaphthene	n.d	n.d	n.d	n.d	0.02	n.d	n.d	0.02	0.71	2.37
Fluorene	0.01	n.d	n.d	0.01	0.07	0.05	0.05	0.05	0.66	2.21
Phenanthrene	n.d	n.d	n.d	n.d	0.07	0.06	0.04	0.05	0.68	2.28
Antrhacene	n.d	0.01	0.01	0.01	0.03	0.02	0.03	0.03	0.65	2.17
Fluoranthene	n.d	n.d	n.d	n.d	0.14	0.16	0.11	0.06	0.69	2.29
Pyrene	n.d	n.d	n.d	n.d	0.13	0.13	0.10	0.12	0.69	2.31
Benzo(a)anthracene	n.d	n.d	n.d	n.d	0.22	0.23	0.15	0.24	0.62	2.08
Chrysene	n.d	n.d	n.d	n.d	0.18	0.20	0.14	0.19	0.69	2.31
Benzo(b)fluoranthene	n.d	n.d	n.d	n.d	0.08	0.10	0.01	0.10	0.61	2.03
Benzo(k)fluoranthene	n.d	n.d	n.d	n.d	0.01	0.01	0.00	0.02	0.62	2.05
Benzo(a)pyrene	n.d	0.16	n.d	0.16	0.11	0.11	0.06	0.11	0.47	1.58
Dibenzo(a,h)anthracene	n.d	n.d	n.d	n.d	n.d	n.d	n.d	n.d	0.57	1.92
Ideno(1,2,3-c,d)pyrene	n.d	0.31	0.31	n.d	n.d	0.07	0.07	0.14	2.62	8.73
Benzo(ghi)perylene	0.17	0.16	0.16	n.d	n.d	n.d	n.d	n.d	1.27	4.25

*C: composition
*n.d : not detected
*LOD: Limits of Detection
*LOD: Limits of Quantification

S19: Mean and total concentrations of PFAS

Table S7. Concentrations of PFAS leached out from unexposed and UV exposed block samples

PFAS	Concentration (ng/L)								IDL (ng/mL)	MDL (ng/L)
	Before UV exposure				After UV exposure					
	C 1	C 2	C 3	C 4	C 1	C 2	C 3	C 4		
PFBA	0.55	0.53	0.23	0.12	0.52	0.55	0.095	0.69	0.03	0.19
PFPeA	0.14	0.19	0.05	0.04	0.20	0.22	0.010	0.24	0.02	0.10
PFHxA	0.40	0.39	0.24	0.03	0.41	0.44	0.239	0.63	0.01	0.05
PFHpA	0.31	0.33	0.22	0.04	0.27	0.27	0.196	0.38	0.01	0.04
PFOA	0.83	0.66	0.30	0.02	0.30	0.30	0.300	0.30	0.01	0.60
PFNA	0.14	0.12	0.11	0.08	0.13	0.11	0.087	0.15	0.01	0.04
PFDA	0.11	0.21	0.06	0.10	0.13	0.06	0.040	0.16	0.01	0.04
PFUdA	0.12	0.36	0.06	0.14	0.05	0.13	0.041	0.17	0.01	0.04
PFBS	0.57	0.84	0.39	0.09	1.42	1.35	0.538	5.32	0.02	0.10
PFHxS	0.08	0.01	0.14	0.06	0.05	0.05	0.045	0.15	0.01	0.09
PFOS	0.47	0.52	0.01	0.06	0.22	0.43	0.069	1.63	0.01	0.07

PFDS	0.41	0.32	0.45	0.08	0.33	0.76	0.388	0.81	0.02	0.11
6:2 FTS	1.85	1.27	1.97	0.20	1.18	1.23	1.920	1.74	0.01	0.20
8:2 FTS	1.63	1.15	2.80	0.41	0.68	0.99	2.917	1.50	0.01	0.03
**ΣPFAS**	**7.6**	**6.9**	**7.0**	**11.8**	**5.9**	**6.9**	**6.9**	**13.7**	**-**	**-**

*C: Composition
*IDL: Instrument Detection Limit
*MDL: Method Detection Limit

S20: Correlation between metals, PFAS leaching Vs material composition (variables) for unaged samples

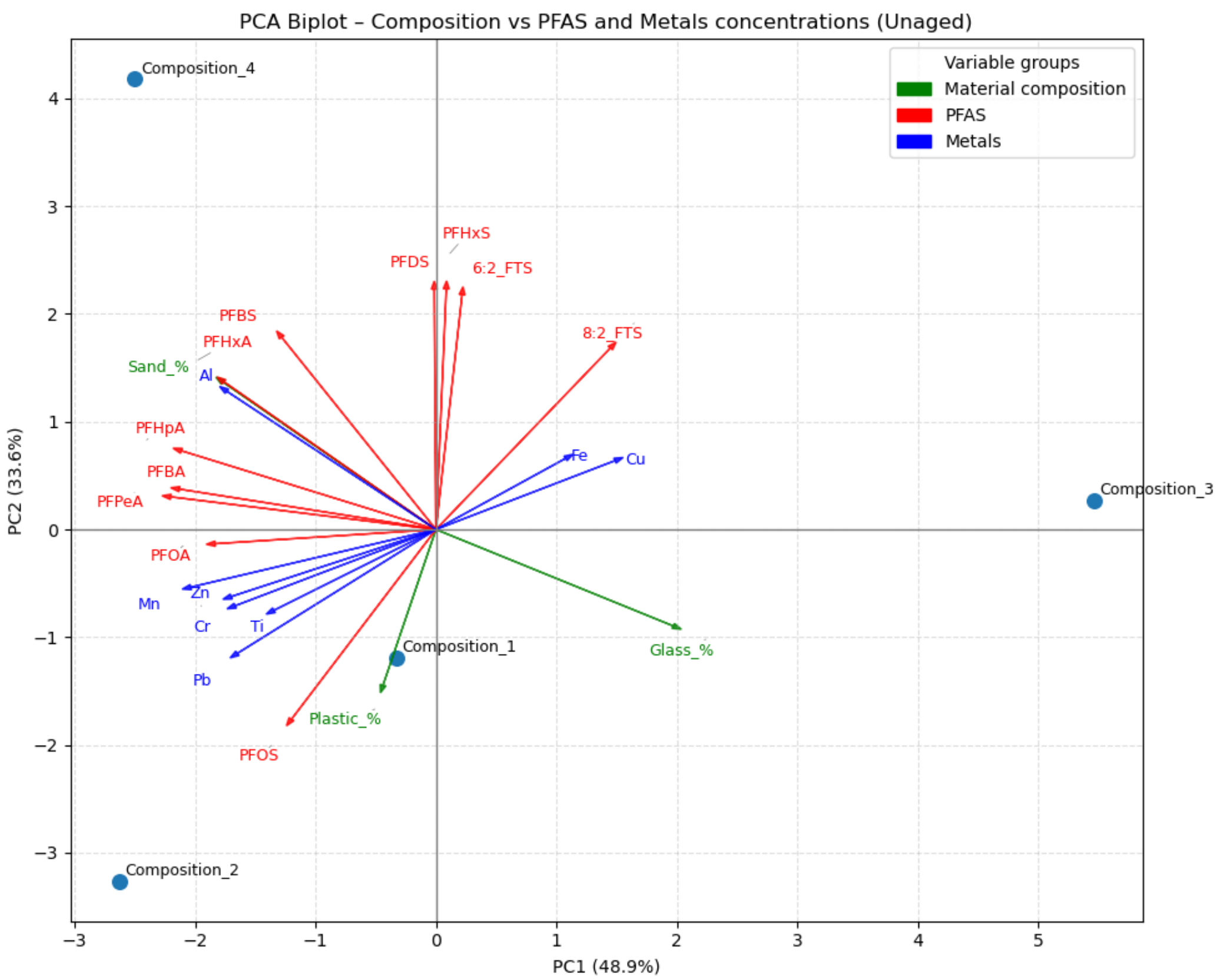

Figure S8. PCA biplot showing relationship between material percentages in composition (green), PFAS (red) and metal (blue) concentrations in the leachates of unaged samples. Compositions are separated along PC1 and PC2 according to differences in PFAS and metals concentrations.

S21: Correlation between material compositions variables with PFAS and metal concentrations in aged and unaged samples (concentration difference was used in this PCA)

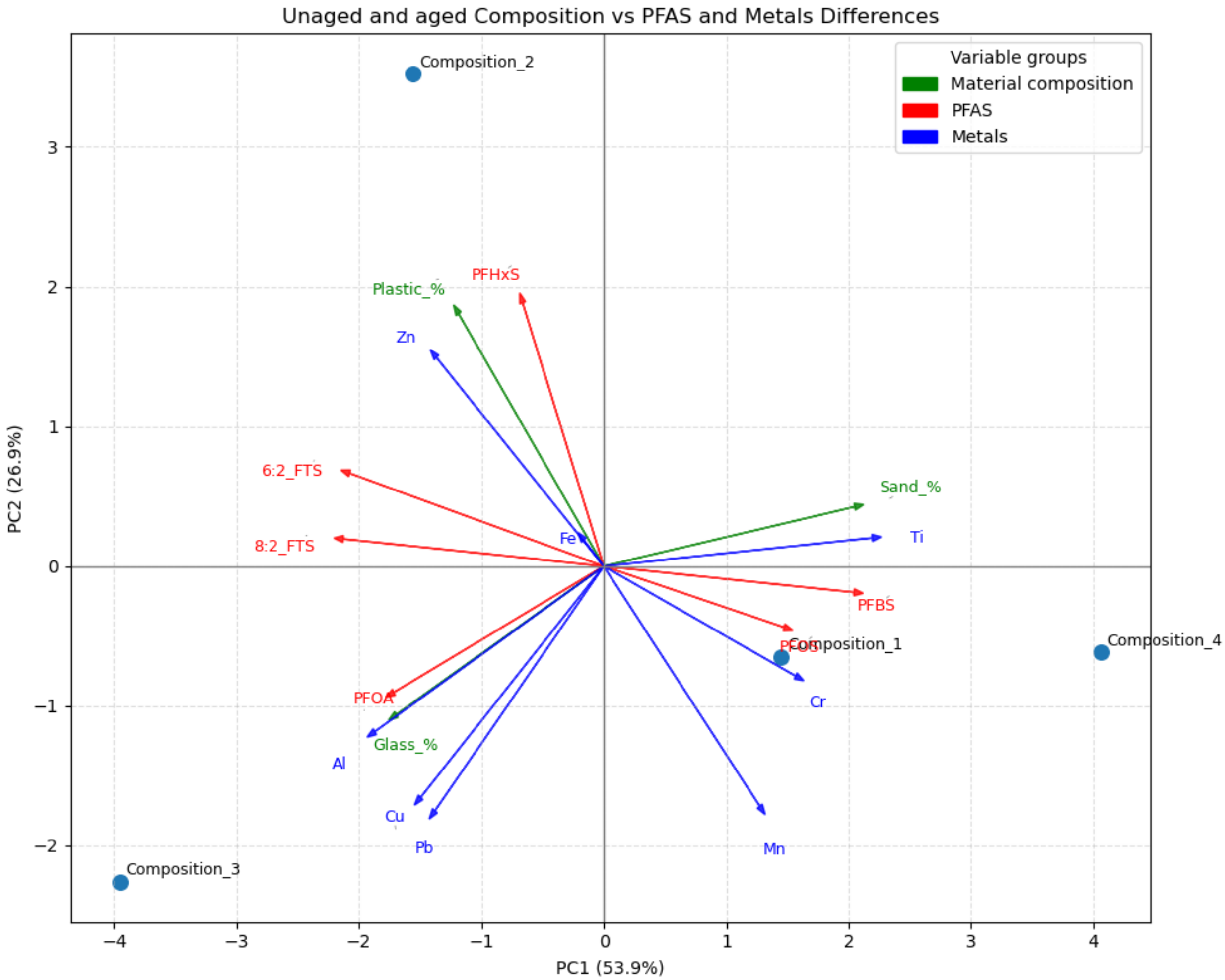

Figure S9. PCA biplot of correlation between the unaged and aged samples material compositions variables vs PFAS and metal concentrations differences. PC1 (53.9%) is based on how strongly aging affected PFAS and metal leaching. PC2 (26.9%) adds secondary variation like distinguishing between PFAS type or metal behavior.

S22: More details on compounds detected during the NTA of the leachates

Table S8. Identification of plastic additives by non-targeted screening in leachate samples

Name	Formula	CAS	rt	mz	Area	ms1_error	Fragments	Adduct_type	Mode
Niacinamide	C6H6N2O1	CAS_RN: 98-92-0	1.29	123.0553	758753	-0.1	{78.03394, 80.04941}	[M+H]+	pos
Laurylethanolamide	C14H29N1O2	CAS_RN: 142-78-9	10.51	244.22729	445755	-0.7	nan	[M+H]+	pos
Benzaldehyde	C7H6O1	CAS_RN: 100-52-7	11.23	107.04913	2572686	0.1	nan	[M+H]+	pos
Galaxolidone	C18H24O2	CAS_RN: 256393-37-0	13.85	273.18505	3577499	-0.5	{157.10118, 197.13309}	[M+H]+	pos
Tris(2-butoxyethyl) phosphate	C18H39O7P1	CAS_RN: 78-51-3	14.37	399.25051	552368	0.3	nan	[M+H]+	pos
Tranexamic acid	C8H15N1O2	CAS_RN: 1197-18-8	4.99	158.11754	1887455	0.1	{123.06657}	[M+H]+	pos
6-Phenyl-1,3,5-triazine-2,4-diamine	C9H9N5	CAS_RN: 91-76-9	5.4	188.09308	2477622	-0.1	{68.02406, 85.05074}	[M+H]+	pos
Scopoletin	C10H8O4	CAS_RN: 92-61-5	6.16	193.04946	1301008	0.4	{121.02835, 150.02682}	[M+H]+	pos
Triethyl citrate	C12H20O7	CAS_RN: 77-93-0	7.51	277.12811	469143	0.3	{139.00302, 157.04937}	[M+H]+	pos
DEET	C12H17N1O1	CAS_RN: 134-62-3	8.69	192.13824	894007	0.2	{105.03352, 119.04917}	[M+H]+	pos
5-Hydroxyculmorin	C15H26O3	CAS_RN: 139680-63-0	9.4	237.18494	643386	-0.1	nan	[M-H2O+H]+	pos
Triphenylphosphine oxide	C18H15O1P1	CAS_RN: 791-28-6	9.64	279.09326	491005	0.3	{201.04666}	[M+H]+	pos

Benzaldehyde, 3-hydroxy-	C7H6O2	CAS_RN: 100-83-4	5.01	121.0297	1643092	-1.7	nan	[M-H]-	neg
Monoethyl phthalate	C10H10O4	CAS_RN: 2306-33-4	5.27	193.05084	824633	-1.1	{121.02945, 121.05524, 149.06075}	[M-H]-	neg
Diethyl phthalate	C12H14O4	CAS_RN: 84-66-2	6.86	221.08223	1677977	-1.4	{121.02942, 177.09202}	[M-H]-	neg
Propylparaben	C10H12O3	CAS_RN: 94-13-3	8.51	179.07152	724950	-0.8	{92.02671, 93.03455}	[M-H]-	neg

S23: Most contributing processes for the three top contributing activities for each impact category

Table S9. Most contributing processes regarding GWP100.

Activity	Process	Contribution to total GWP100
Extrusion of material mixture	Heat production, at hard coal industrial furnace 1-10MW	20.54% - 21.71%
Processing of waste plastic	Treatment of municipal solid waste, sanitary landfill	12.44% - 13.10%
Drying the recycled PET	Heat production, at hard coal industrial furnace 1-10MW	6.88% - 10.74%

Table S10. Most contributing processes regarding HTPinf for composition 1 and 4.

Activity	Process	Contribution to total HTPinf
Processing of waste plastic	Treatment of brake wear emissions, lorry	15.33% - 15.83%
Extrusion of material mixture	Treatment of hard coal ash, residual material landfill	15.32% - 15.88%
Mixing the raw materials	Treatment of hard coal ash, residual material landfill	1.99% - 2.00%

Table S11. Most contributing processes regarding HTPinf for composition 2 and 3.

Activity	Process	Contribution to total HTPinf
Processing of waste plastic	Treatment of brake wear emissions, lorry	15.33% - 15.83%
Extrusion of material mixture	Treatment of hard coal ash, residual material landfill	15.32% - 15.88%
Drying the recycled PET	Treatment of hard coal ash, residual material landfill	3.73% - 3.77%

Table S12. Most contributing processes regarding METPinf.

Activity	Process	Contribution to total METPinf
Processing of waste plastic	Treatment of brake wear emissions, lorry	25.16% - 25.63%
Extrusion of material mixture	Natural gas production, unprocessed, at extraction	12.44% - 13.10%
Production of vegetable oil	Seed-cotton production, conventional	6.88% - 10.74%

Table S13. Most contributing processes regarding PMFP.

Activity	Process	Contribution to total PMFP
Extrusion of material mixture	Electricity production, lignite	17.42% - 18.03%
Processing of waste plastic	Electricity production, lignite	16.78% - 17.29%
Drying the recycled PET	Heat production, at hard coal industrial furnace 1-10 MW	4.48% - 7.12%

References

1. Bilek JGeorge, Kollonitsch Valerie, Kline CH. ZINC CHEMICALS IN PLASTICS SYSTEMS. *Ind Eng Chem*. 1966;58(5):28-36. doi:10.1021/ie50677a008
2. Peng G, Pu Z, Chen F, et al. Metal leaching from plastics in the marine environment: An ignored role of biofilm. *Environ Int*. 2023;177:107988. doi:10.1016/j.envint.2023.107988
3. Cheng X, Forsythe J, Peterkin E. Some factors affecting SPME analysis and PAHs in Philadelphia's urban waterways. *Water Res*. 2013;47(7):2331-2340. doi:10.1016/j.watres.2013.02.006
4. Chambers MC, Maclean B, Burke R, et al. A cross-platform toolkit for mass spectrometry and proteomics. *Nat Biotechnol*. 2012;30(10):918-920. doi:10.1038/nbt.2377
5. Hollender J, Schymanski EL, Ahrens L, et al. NORMAN guidance on suspect and non-target screening in environmental monitoring. *Environ Sci Eur*. 2023;35(1):75. doi:10.1186/s12302-023-00779-4
6. Horai H, Arita M, Kanaya S, et al. MassBank: a public repository for sharing mass spectral data for life sciences. *Journal of Mass Spectrometry*. 2010;45(7):703-714. doi:10.1002/jms.1777
7. Amran T, Mohd T, Bohairah NA, Jaafar MZ. Characterization of Quartz Sand as an Adsorbent for Anionic Surfactant Adsorption with Presences of Alkaline and Polymer. *Chem Eng Trans*. 2022;95:283-288. doi:10.3303/CET2295048
8. Ramasamy V, Govindasamy S, Meenakshisundaram V, Ponnusamy V. *DISTRIBUTION AND CHARACTERIZATION OF MINERALS AND NATURALLY OCCURRING RADIONUCLIDES IN RIVER SEDIMENTS*. Vol 5. 2010.
9. Chandrasekaran A, Senthil Kumar CK, Sathish V, Manigandan S, Tamilarasi A. Effect of minerals and heavy metals in sand samples of Ponnai river, Tamil Nadu, India. *Sci Rep*. 2021;11(1):23199. doi:10.1038/s41598-021-02717-x
10. Petrovič A, Čolnik M, Prša A, et al. Comparative Analysis of Virgin and Recycled Thermoplastic Polymer based on Thermochemical Characteristics. *Chem Eng Trans*. 2022;94:1321-1326. doi:10.3303/CET2294220
11. Chaudhary AK, Vijayakumar RP. Studies on biological degradation of polystyrene by pure fungal cultures. *Environ Dev Sustain*. 2020;22(5):4495-4508. doi:10.1007/s10668-019-00394-5
12. Goel AK, Kurek J, Bartkowiak G, et al. *New Advances in Materials Science and Engineering Vol. 1*. (Goel DrAK, ed.). Book Publisher International (a part of SCIENCEDOMAIN International); 2019. doi:10.9734/bpi/namse/v1
13. Doğan M. Ultraviolet light accelerates the degradation of polyethylene plastics. *Microsc Res Tech*. 2021;84(11):2774-2783. doi:10.1002/jemt.23838
14. Martínez JL, Rodríguez Rego JM, Cerezo LM, Madrigal MDS, Macías-García A. Odour characterisation of recycled HDPE in different washing and processing processes. *Environmental Science and Pollution Research*. 2024;31(47):58136-58151. doi:10.1007/s11356-024-34976-2
15. Bhattarai B, Kusano Y, Cederberg TL, Jensen LK, Granby K, Pedersen GA. Chemical characterization of virgin and recycled polyethylene terephthalate films used for food contact applications. *European Food Research and Technology*. 2024;250(2):533-545. doi:10.1007/s00217-023-04400-z

16. Worku BG, Wubieneh TA. Mechanical Properties of Composite Materials from Waste Poly(ethylene terephthalate) Reinforced with Glass Fibers and Waste Window Glass. *Int J Polym Sci*. 2021;2021:1-14. doi:10.1155/2021/3320226
17. Imoisili PE, Ukoba KO, Jen TC. Synthesis and characterization of amorphous mesoporous silica from palm kernel shell ash. *Boletín de la Sociedad Española de Cerámica y Vidrio*. 2020;59(4):159-164. doi:10.1016/j.bsecv.2019.09.006
